# Mechanism-defined secondary hypertension enables precision diagnosis and treatment: an evidence review

**DOI:** 10.3389/fcvm.2026.1958980

**Published:** 2026-10-14

**Authors:** Arbind Kumar Choudhary, Anamitra Hait, Maheshkumar Baladaniya

**Affiliations:** 1 Department of Pharmacology, Government Erode Medical College and Hospital, Perundurai, Tamil Nadu, India; 2 Department of Internal Medicine, Eastern Railway Production Unit Hospital, Chittaranjan, West Bengal, India; 3 Department of Physical Therapy, Neighborhood PT PC, New City, NY, United States

**Keywords:** endocrine hypertension, molecular diagnostics, precision medicine, primary aldosteronism, renovascular hypertension, resistant hypertension, secondary hypertension, targeted therapy

## Abstract

**Background:**

Secondary hypertension is under-recognised despite the availability of mechanism-based diagnostic tests and, for several aetiologies, potentially curative or highly targeted therapy. The clinically important question is not only which causes exist, but how reliably mechanisms translate into diagnosis and cause-directed treatment.

**Objectives:**

To systematically synthesise evidence across the principal causes of secondary hypertension using a mechanism → diagnosis → therapy framework; to compare diagnostic performance and cause-directed treatment effects; and to examine concordance between contemporary society guidelines and real-world implementation.

**Methods:**

This systematic review followed Preferred Reporting Items for Systematic Reviews and Meta-Analyses (PRISMA) 2020, with diagnostic accuracy reporting informed by PRISMA-DTA. MEDLINE (PubMed), Embase, CENTRAL, Web of Science Core Collection, Scopus, ClinicalTrials.gov, and the WHO ICTRP were searched from inception to 30 June 2025, supplemented by citation searching. The formal search identified 580 records; after removal of 168 duplicates, 412 records were screened, 106 full-text reports were assessed, and 58 included evidence records were represented across 63 publications. The review was not prospectively registered in PROSPERO. Screening, extraction and structured appraisal were performed by a single reviewer (the first author); therefore, no dual-reviewer agreement statistic was applicable. Risk of bias was appraised with RoB 2, ROBINS-I, QUADAS-2, and AMSTAR 2 as appropriate, and certainty was summarised using GRADE. A targeted post-search surveillance update through 31 July 2026 was used only to contextualise major newly available guidelines and Phase 3 trial reports and did not alter the original PRISMA counts.

**Results:**

Primary aldosteronism (PA) is best understood as a continuum of renin-independent aldosterone excess: in Brown et al. [The unrecognized prevalence of primary aldosteronism: a cross-sectional study. *Ann Intern Med*. (2020) **173**:10–20], biochemically overt autonomous aldosterone production was present in 11.3%, 15.7%, 21.6%, and 22.0% across normotensive, stage 1, stage 2, and resistant-hypertension strata, respectively. In unilateral PA, the PASO cohort distinguished complete biochemical success after adrenalectomy (94%; 656/699) from complete clinical success (37%; 259/705), with partial clinical improvement in a further 47%. PATHWAY-2 established spironolactone as the most effective fourth-line drug in resistant hypertension; CORAL, ASTRAL, STAR, and DRASTIC did not support routine revascularisation for stable atherosclerotic renal-artery stenosis; plasma-free metanephrines remained highly sensitive for phaeochromocytoma/paraganglioma; and PA testing in eligible patients with resistant hypertension remained only 1.6%–2.1%. Post-search Phase 3 trials of selective aldosterone synthase inhibition showed approximately 9 mm Hg placebo-corrected office systolic blood pressure reductions, but cardiovascular-outcome benefit has not been established, and electrolyte/renal monitoring remains necessary.

**Conclusions:**

Across secondary hypertension, a limited set of pathophysiological effector pathways maps to specific diagnostic tests and cause-directed treatments. The strongest opportunities for clinical gain are earlier recognition of high-yield phenotypes, accurate aetiological subtyping, and implementation of established testing pathways; emerging aldosterone synthase inhibitors expand blood-pressure-lowering options but should not yet be interpreted as proven cardiovascular-outcome therapies.

## 1 Introduction

Raised blood pressure is the single largest modifiable cause of premature cardiovascular death, and its global burden continues to grow. The number of adults living with hypertension roughly doubled between 1990 and 2019, and treatment and control remain inadequate across most of the world (1). Earlier syntheses established the scale of the problem and its uneven distribution (2), and later population analyses confirmed that prevalence has shifted towards low- and middle-income settings while control rates lag furthest behind (3). Against this vast denominator, a specific and potentially curable cause can be identified in a clinically meaningful minority. Yet secondary hypertension is repeatedly missed, in part because the phenotypes that should prompt investigation—young-onset, resistant, or abruptly worsening disease, spontaneous or diuretic-induced hypokalaemia, an adrenal mass, or disproportionate target-organ damage—are not reliably acted upon. Rimoldi et al. set out a practical framework for when, in whom, and how to screen, and that framework still frames contemporary practice (4). The stakes of recognition are substantial. A cause identified early can normalise blood pressure with a targeted intervention, avert years of receptor- and pressure-mediated target-organ damage, and reduce a lifelong burden of polypharmacy; conversely, a cause missed condemns the patient to sustained uncontrolled pressure and, in aldosteronism in particular, to harm that exceeds what the blood-pressure reading alone would predict. The phenotypes that should prompt investigation are therefore not academic curiosities but actionable clinical signals, and the central practical failure of the field is not an absence of tests but a failure to deploy them in the patients most likely to benefit.

Resistant hypertension is the phenotype in which secondary causes concentrate. The American Heart Association's (AHA) scientific statements define it as blood pressure above goal despite three appropriately dosed antihypertensive agents, including a diuretic, or controlled blood pressure requiring four or more drugs, and set out its evaluation (5, 6). Population estimates place apparent treatment-resistant hypertension near one in eight treated adults in the United States (7), with comparable incidence and adverse prognosis in integrated-care cohorts (8). Ambulatory monitoring further shows that a substantial proportion of apparent resistance reflects a white-coat effect rather than true resistance, refining the population in whom secondary work-up is warranted (9). Resistance therefore functions less as a diagnosis than as an enrichment strategy: it raises the pre-test probability of an identifiable cause and should trigger structured investigation. The corollary is operational: a patient who remains above target on three drugs, including a diuretic, or who requires four or more agents for control has crossed a threshold at which the yield of investigation justifies its cost, and at which a failure to investigate is itself a clinical decision with consequences.

Guidance for this population is now delivered through living, frequently updated documents. The 2025 AHA/ACC guideline of Jones and colleagues is the current United States reference standard for detection, evaluation and management of high blood pressure in adults (10); the 2024 European Society of Cardiology (ESC) guideline (11) and the 2023 European Society of Hypertension (ESH) guideline (12) provide the contemporary European positions, and the 2020 International Society of Hypertension guidelines offer a resource-stratified global standard (13). The 2017/2018 ACC/AHA guideline (14) and the 2018 ESC/ESH guideline (15) are historical and are retained here only for concordance mapping, while the World Health Organization's 2021 pharmacological guidance addresses drug treatment in resource-variable settings (16).

The central tension of this review is a genuine divergence in screening philosophy. The 2025 Endocrine Society guideline on primary aldosteronism (PA) suggests, for the first time, that screening be considered for essentially all hypertension (17)—a substantial shift from the high-risk, case-finding approach of the 2016 Endocrine Society guideline (18) and its 2008 predecessor (19). Cardiology and hypertension guidelines have not adopted a screen-all approach and retain targeted case detection (10–13). This guideline-vs.-guideline discordance sits alongside, and is dwarfed by, the discordance between any guideline and actual testing rates, developed in Section 3.

Cause-specific society documents complete the diagnostic scaffolding. Dedicated guidelines address phaeochromocytoma and paraganglioma (PPGL) (20), the diagnosis (21) and treatment (22) of Cushing's syndrome, fibromuscular dysplasia (23), the joint respiratory–hypertension management of obstructive sleep apnoea (24), and the role of catheter-based renal denervation (25). Together, these define the tests and treatments that a precision approach must deploy in the right patient.

Accordingly, the review question, framed as PICO, is in adults with hypertension (population), which secondary causes—primary aldosteronism, renovascular disease and fibromuscular dysplasia, obstructive sleep apnoea, phaeochromocytoma and paraganglioma, Cushing's syndrome, and monogenic hypertension (conditions/exposures)—have extractable evidence on mechanism, diagnostic accuracy and cause-directed therapy (outcomes), and how concordant are current society guidelines with one another and with practice (comparison)? This systematic review therefore separates the formal PRISMA evidence set from later contextual surveillance, avoids *de novo* pooling where studies are clinically or methodologically incompatible, and uses Tables 1–8 and Figures 1–8 to link evidence provenance to the mechanism → diagnosis → therapy framework. The complete structured extraction dataset is provided as Supplementary Material.

**Table 1:** Characteristics of key included evidence records.

First author (year)	Design	Setting/N	Aetiology	Key extractable result	RoB tool/rating	GRADE	References
Rossi (2006)	Prospective	Italy, 14 centres, *n* = 1,125	Primary aldosteronism	Prevalence 11.2%; APA 4.8%; IHA 6.4%	ROBINS-I, Moderate	Moderate	(34)
Monticone (2017)	Prospective	Primary care, *n* = 1,672	Primary aldosteronism	5.9% overall; 3.9% → 11.8% grade 1 → 3	ROBINS-I, Moderate	Moderate	(35)
Brown (2020)	Cross-sectional	USA	Primary aldosteronism	Unrecognised renin-independent aldosteronism across all BP categories	ROBINS-I, Moderate	Moderate	(36)
Käyser (2016)	MA/meta-regression	39 studies	Primary aldosteronism	Method and setting explain the prevalence range	AMSTAR-2, Low–moderate	Moderate	(37)
Douma (2008)	Retrospective	Resistant HTN, *n* = 1,616	Primary aldosteronism	11.3% (5.5%–20% by series)	ROBINS-I, Moderate	Moderate	(38)
Xu (2020)	Prospective	China, newly diagnosed, *n* = 2,046	Primary aldosteronism	≥4%	ROBINS-I, Moderate	Moderate	(39)
Monticone (2018)	Meta-analysis	31 studies; 3,838 PA/9,284 EH	Primary aldosteronism	Stroke OR 2.58; AF 3.52; HF 2.05; CAD 1.77	AMSTAR-2, Low–moderate	Moderate	(63)
Hundemer (2018)	Retrospective	Medically treated PA	Primary aldosteronism	Residual CV risk when renin remains suppressed	ROBINS-I, Moderate	Low–moderate	(66)
Choi (2011)	Mechanistic	APA tissue	Primary aldosteronism	*KCNJ5* somatic mutations	N/A	N/A (mechanism)	(51)
Williams (2017), PASO	Cohort	*n* = 705, 12 centres	PA (surgery)	Complete clinical success 37% (259/705); partial clinical success 47% (334/705); complete biochemical success 94% (656/699)	ROBINS-I, Moderate	Low–moderate	(74)
Yang (2025), PAMO	Cohort	*n* = 1,258	PA (medical)	Unsuppressed renin = biochemical success	ROBINS-I, Moderate	Low–moderate	(75)
Dekkers (2016), SPARTACUS	RCT	*n* = 184	PA (diagnosis)	CT- vs. AVS-guided therapy: no 1-year difference in drug intensity	RoB 2, Some concerns	Moderate	(69)
Wu (2023), MATCH	RCT	*n* = 143	PA (diagnosis)	[^11^C]metomidate PET ≈ AVS; 77/78 surgical success	RoB 2, Low–some concerns	Moderate	(72)
Williams (2015), PATHWAY-2	Crossover RCT	*n* = 314	Resistant HTN	Spironolactone vs. placebo −8.70 mm Hg (−9.72 to −7.69)	RoB 2, Low	High	(104)
Krieger (2018), ReHOT	RCT	*n* = 187	Resistant HTN	Spironolactone superior to clonidine	RoB 2, Low–some concerns	High	(106)
Cooper (2014), CORAL	RCT	*n* = 947	Renal-artery stenosis	Composite HR 0.94 (0.76–1.17)	RoB 2, Low	Moderate	(88)
ASTRAL (2009)	RCT	*n* = 806	Renal-artery stenosis	No benefit of revascularisation vs. medical therapy	RoB 2, Some concerns	Moderate	(89)
Bax (2009), STAR	RCT	*n* = 140	Renal-artery stenosis	No benefit on eGFR decline	RoB 2, Some concerns	Moderate	(90)
van Jaarsveld (2000), DRASTIC	RCT	*n* = 106	Renal-artery stenosis	Angioplasty vs. medical: no durable BP benefit	RoB 2, Some concerns	Moderate	(91)
Martínez-García (2013), HIPARCO	RCT	*n* = 194	OSA + resistant HTN	24-h mean BP −3.1 mm Hg (0.6–5.6); improved dipping	RoB 2, Low–some concerns	Moderate	(83)
McEvoy (2016), SAVE	RCT	*n* = 2,717	OSA + CVD	CV HR 1.10 (0.91–1.32); adherence 3.3 h/night	RoB 2, Low	Moderate	(84)
Pedrosa (2011)	Prospective	*n* = 125	Resistant HTN	OSA in 64%, commonest secondary cause	ROBINS-I, Moderate	Moderate	(81)
Lenders (2002)	Diagnostic accuracy	*n* = 858 (214 PPGL)	PPGL	Plasma-free metanephrines Se 99%, Sp 89%	QUADAS-2, Low–some concerns	Moderate–high	(94)
Han (2019)	Diagnostic accuracy MA	PPGL	PPGL	^68^Ga-DOTA-SSTR PET, per-lesion 92%–100%	AMSTAR-2, Low–some concerns	Moderate–high	(95)
Bhatt (2014), HTN-3	Sham RCT	*n* = 535	Renal denervation	No significant office-SBP difference vs. sham	RoB 2, Low	Moderate	(107)
Böhm (2020), SPYRAL OFF-MED	Sham RCT	Off medication	Renal denervation	24-h SBP −3.9 mm Hg vs. sham	RoB 2, Low–some concerns	Moderate	(111)
Azizi (2023), RADIANCE II	Sham RCT	*n* = 224	Renal denervation	Daytime ambulatory SBP −6.3 mm Hg	RoB 2, Low–some concerns	Moderate	(115)
Kario (2022), REQUIRE	Sham RCT	*n* = 136	Renal denervation	Negative (East Asia)	RoB 2, Low–some concerns	Moderate	(117)
Cohen (2021)	Retrospective	VHA, *n* = 269,010	Implementation	1.6% of eligible resistant HTN tested for PA	ROBINS-I, Moderate	Moderate	(119)
Jaffe (2020)	Retrospective	Kaiser	Implementation	2.1% tested	ROBINS-I, Moderate	Moderate	(120)
Milliez (2005)	Cohort	PA vs. controls	Primary aldosteronism	Increased rate of cardiovascular events vs. essential HTN	ROBINS-I, Moderate	Low–moderate	(64)
Ohno (2018), JPAS	Multicentre cohort	Japan	Primary aldosteronism	Excess cardiovascular disease and target-organ damage	ROBINS-I, Moderate	Moderate	(65)
Hundemer (2018)	Retrospective	Medically/surgically treated PA	Primary aldosteronism	Renal outcomes differ by treatment modality	ROBINS-I, Moderate	Low–moderate	(67)
Rossi (2012), AVIS	International multicentre	AVS registry	PA (subtyping)	AVS success centre-dependent; complications ∼0.61%	ROBINS-I, Moderate	Moderate	(70)
Mahfoud (2022), SPYRAL ON-MED 36 months	Sham RCT, long-term	On medication, 36-month follow-up	Renal denervation	Office SBP −10.0 mm Hg at 36 months	RoB 2, Low–some concerns	Moderate	(112)
Azizi (2021), RADIANCE-HTN TRIO	Sham RCT	Triple-pill background	Renal denervation	Daytime ambulatory SBP −4.5 mm Hg vs. sham	RoB 2, Low–some concerns	Moderate	(114)
Bratton (2015)	Systematic review/MA	CPAP vs. MAD	OSA	CPAP: SBP −2.5 mm Hg (1.5–3.5); DBP −2.0 (1.3–2.7)	AMSTAR-2, Low–some concerns	Moderate	(85)
Peppard (2000)	Prospective cohort	Wisconsin Sleep Cohort	OSA	Dose-dependent incident hypertension	ROBINS-I, Moderate	Moderate	(79)
Nieto (2000)	Cross-sectional	Sleep Heart Health Study	OSA	Sleep-disordered breathing associated with hypertension	ROBINS-I, Moderate	Moderate	(80)
Logan (2001)	Cross-sectional	Drug-resistant HTN	OSA	83% unrecognised OSA	ROBINS-I, Moderate	Moderate	(82)
Olin (2012)	Registry	US FMD registry, *n* = 447	Fibromuscular dysplasia	∼91% female	ROBINS-I, Moderate	Moderate	(92)

APA, aldosterone-producing adenoma; IHA, idiopathic hyperaldosteronism; EH, essential hypertension; PA, primary aldosteronism; PPGL, phaeochromocytoma and paraganglioma; AVS, adrenal vein sampling; BP, blood pressure; SBP/DBP, systolic/diastolic BP; CV, cardiovascular; OSA, obstructive sleep apnoea; MA, meta-analysis; OR, odds ratio; HR, hazard ratio; Se, sensitivity; Sp, specificity; AF, atrial fibrillation; RCT, randomised controlled trial; CVD, cardiovascular disease; CAD, coronary artery disease; MAD, mandibular advancement device.

Designs, sample sizes, and effect estimates are taken from the published record; values not specified in the source are omitted rather than estimated. Table 1 presents the key evidence records used in the main synthesis; the complete 58-record extraction is supplied in the Supplementary Material. Methods papers (26–33, 121) are reporting/appraisal references rather than clinical evidence rows.

**Table 2:** Mechanisms by aetiology—effector axis, genes, blood-pressure effect, and precision implication.

Aetiology	Effector axis	Molecular/cellular detail	Key genes	Downstream BP effect	Precision implication	References
Primary aldosteronism	RAAS/mineralocorticoid receptor	Somatic *KCNJ5*, *CACNA1D*, *ATP1A1*, *ATP2B3* → zona glomerulosa Ca^2^⁺ influx → *CYP11B2*; germline FH-I–IV; age-related aldosterone-producing cell clusters	*KCNJ5*, *CACNA1D*, *ATP1A1*, *ATP2B3*, *CYP11B2*, *CYP11B1*	Na⁺/volume retention, MR-mediated fibrosis, hypokalaemia, excess AF/stroke/HF vs. EH	Genotype and *CYP11B2* imaging (metomidate PET) inform surgery; unsuppressed renin marks effective MRA (PAMO)	(43, 45, 51–55, 72, 74, 75)
Renovascular (atherosclerotic/FMD)	RAAS/vascular	Stenosis → renin → angiotensin II/aldosterone; FMD is non-atherosclerotic dysplasia, female-predominant	Renin pathway; FMD polygenic	Vasoconstriction plus volume; flash pulmonary oedema if bilateral or solitary kidney	Do not routinely stent atherosclerotic RAS; for focal renal FMD, angioplasty without stent	(23, 86–93)
Obstructive sleep apnoea	Sympathetic/chemoreflex	Intermittent hypoxia → carotid chemoreflex, muscle sympathetic nerve activity, reactive oxygen species, endothelial dysfunction	Catecholamine/ROS pathways (not monogenic)	Nocturnal, non-dipping, and resistant hypertension	Adherence and the resistant HTN phenotype predict BP response (HIPARCO vs. SAVE)	(76–85)
PPGL	Sympathetic/catecholamine	Cluster 1 pseudohypoxia (SDHx, VHL, EPAS1) vs. cluster 2 kinase signalling (RET, NF1, MAX, TMEM127); SDHB confers metastatic risk	*SDHB*, *SDHD*, *VHL*, *RET*, *NF1*, *MAX*, *TMEM127*, *EPAS1*	Paroxysmal or sustained hypertension; catecholamine crisis	Germline cluster guides surveillance and SSTR vs. FDG PET; plasma-free metanephrines first-line	(20, 94–98)
Cushing's syndrome	Cortisol/MR occupancy	Cortisol excess saturates 11*β*-HSD2 → illicit MR occupancy by cortisol	*HSD11B2*, *POMC*/ACTH, *USP8*	Volume plus vasoconstriction; adverse metabolic phenotype	Cause-directed resection; steroid metabolome adjunctive for subtyping	(21, 22, 99–103)
Monogenic	Renal ion transport/MR	ENaC gain of function (Liddle); WNK–KLHL3–CUL3 (Gordon); *CYP11B1/2* chimaera (GRA); MR S810L	*SCNN1B/G*, *WNK1/4*, *KLHL3*, *CUL3*, *CYP11B1/2*, *NR3C2*	Constitutive Na⁺ reabsorption, suppressed renin, electrolyte fingerprint	Amiloride (Liddle), thiazide (Gordon), glucocorticoid (GRA); never MRA-first in Liddle	(56–61)
Resistant HTN (syndrome)	Volume/MR/ENaC	PATHWAY-2 mechanisms: low-renin, volume-expanded; amiloride ≈ spironolactone	Renin as biomarker	Uncontrolled BP on ≥3 drugs including a diuretic	MRA (or amiloride) fourth-line; then phenotype-directed work-up; RDN after work-up	(5, 81, 104–106, 119, 120)

ACTH, adrenocorticotropic hormone; RAAS, renin–angiotensin–aldosterone system; MR, mineralocorticoid receptor; MRA, MR antagonist; ENaC, epithelial sodium channel; GRA, glucocorticoid-remediable aldosteronism; SSTR, somatostatin receptor; RDN, renal denervation; ROS, reactive oxygen species; FDG, fluorodeoxyglucose.

**Table 3:** Diagnostic accuracy of index tests.

Aetiology	Index test	Reference standard	Verified performance	GRADE	References
Primary aldosteronism	Aldosterone-to-renin ratio	Confirmatory suppression tests	Screening test; performance varies by assay, cut-off, posture, drugs and setting	Moderate	(17, 18, 36, 37)
Primary aldosteronism	Adrenal vein sampling (strategy)	Surgical/biochemical outcome	SPARTACUS: no 1-year difference vs. CT; AVIS success centre-dependent; complications ∼0.61%	Moderate	(69–71)
Primary aldosteronism	[^11^C]metomidate PET–CT	Postoperative remission; AVS	MATCH (*n* = 143): PET ≈ AVS; 77/78 surgical success	Moderate	(72, 73)
Primary aldosteronism	LC–MS/MS steroid profiling	AVS/histology	Improves subtyping; identifies 18-oxocortisol and glucocorticoid cosecretion	Moderate	(68)
PPGL	Plasma-free metanephrines	Histology/biochemistry	Se 99% (211/214); Sp 89% (586/644)	Moderate–high	(94)
PPGL	^68^Ga-DOTA-SSTR PET	Composite imaging/histology	Per-lesion 92%–100%; superior in SDHx and metastatic disease	Moderate–high	(95)
Cushing's syndrome	LNSC + UFC + 1 mg DST; steroid metabolome	Multimodal (IPSS, imaging, histology)	Two concordant screening tests before imaging	Moderate	(21, 101, 102)
Renovascular/FMD	Doppler/CTA/MRA	Catheter angiography	FMD string-of-beads diagnostic in the right phenotype; anatomical RAS ≠ physiological significance	Moderate	(23, 92, 93)
OSA	Polysomnography (AHI)	Sleep-study scoring	Reference standard; yield in resistant HTN 64%–83%	Moderate	(81, 82)
Monogenic	Targeted panel (ENaC, WNK, KLHL3, CUL3, *CYP11B1/2*, *NR3C2*)	Pathogenic variant + electrolyte/renin fingerprint	Diagnostic in young-onset, familial, or pathognomonic electrolyte phenotypes	Low–moderate	(56–60)

LNSC, late-night salivary cortisol; UFC, urinary-free cortisol; DST, dexamethasone suppression test; IPSS, inferior petrosal sinus sampling; CTA/MRA, CT/MR angiography; AHI, apnoea–hypopnoea index; LC–MS/MS, liquid chromatography–tandem mass spectrometry.

**Table 4:** Cause-directed treatment outcomes.

Aetiology	Intervention	Comparator	Verified result	Certainty	Refs
PA, unilateral	Subtype-selected adrenalectomy	MRA	PASO: complete biochemical success 94% (656/699); complete clinical success 37% (259/705); partial clinical success 47% (334/705)	Low–moderate	(74)
PA, bilateral/medical	MRA, renin-guided (PAMO)	Non-targeted antihypertensives	Unsuppressed renin defines effective therapy (*n* = 1,258); residual suppressed renin → excess CV/renal risk	Low–moderate	(66, 67, 75)
Resistant HTN	Spironolactone 25–50 mg (fourth drug)	Bisoprolol, doxazosin, placebo; clonidine	−8.70 mm Hg vs. placebo (−9.72 to −7.69); ReHOT concordant; amiloride ≈ spironolactone	High	(104–106)
Atherosclerotic RAS	Renal-artery stent + medical therapy	Medical therapy alone	No composite CV/renal benefit (CORAL HR 0.94, 0.76–1.17); ASTRAL, STAR, DRASTIC concordant	Moderate	(88–91)
OSA (resistant HTN)	CPAP	Usual care	HIPARCO: 24-h mean BP −3.1 mm Hg (0.6–5.6); improved dipping	Moderate	(83)
OSA (CVD, unselected)	CPAP	Usual care	SAVE: HR 1.10 (0.91–1.32); mean adherence 3.3 h/night	Moderate	(84)
Uncontrolled/resistant HTN after work-up	Catheter-based renal denervation	Sham	HTN-3 negative; second-generation −3.9 to −7.4 mm Hg; 36-month ON-MED −10.0; REQUIRE negative	Moderate	(25, 107–117)
Liddle's syndrome	Amiloride (ENaC blocker)	Non-targeted, including MRA	Mechanism-specific control; spironolactone ineffective	Low	(57)
Gordon's syndrome/PHAII	Thiazide	Non-targeted	Corrects NCC over-activation; treats hypertension and hyperkalaemia	Low	(58, 59)
GRA/FH-I	Low-dose glucocorticoid	Non-targeted	Suppresses ACTH-driven chimeric aldosterone synthase	Low	(56)
Cushing's syndrome	Cause-directed resection	Medical therapy/radiotherapy	First-line in expert centres	Low–moderate	(22, 102)
PPGL	*α*-blockade then resection; cluster-guided follow-up	Unprepared surgery	Perioperative α-blockade standard; SDHB intensifies surveillance	Moderate	(20, 96–98)

NCC, sodium-chloride cotransporter; PHAII, pseudohypoaldosteronism type II; FH-I, familial hyperaldosteronism type I.

The −7.4 mm Hg figure denotes the upper bound of second-generation sham-controlled ambulatory reductions across the RADIANCE and SPYRAL programmes.

**Table 5:** GRADE summary of principal findings.

No.	Outcome	Design/N	RoB	Inconsistency	Indirectness	Imprecision	Publication bias	Effect	Certainty	References
1	Spironolactone as fourth drug, home SBP	1 crossover RCT (PATHWAY-2, *n* = 314) + ReHOT	Low	Not serious (ReHOT concordant)	Not serious	Not serious	Undetected	−8.70 mm Hg vs. placebo (−9.72 to −7.69)	High	(104, 106)
2	Renal stenting vs. medical therapy (CV/renal composite)	4 RCTs (DRASTIC, STAR, ASTRAL, CORAL)	Low–some	Not serious	Serious (anatomical, not physiological, selection)	Not serious	Undetected	CORAL HR 0.94 (0.76–1.17); no benefit	Moderate	(88–91)
3	CPAP blood-pressure effect in resistant HTN	RCT (HIPARCO, *n* = 194)	Low–some	Serious vs. unselected CPAP trials	Not serious	Some	Suspected (small positive trials)	24-h mean BP −3.1 mm Hg (0.6–5.6)	Moderate	(83)
4	CPAP for CV events (unselected OSA + CVD)	RCT (SAVE, *n* = 2,717)	Low	n/a (single large RCT)	Serious (adherence 3.3 h; not resistant HTN)	Not serious	Undetected	HR 1.10 (0.91–1.32)	Moderate	(84)
5	Adrenalectomy clinical cure (unilateral PA)	PASO cohorts (*n* = 705, 12 centres)	Moderate	Not serious for biochemical success; clinical success heterogeneous by centre	Not serious	Not serious	Suspected	Complete biochemical success 94% (656/699); complete clinical success 37% (259/705); partial clinical success 47% (334/705)	Low–moderate	(74)
6	Plasma-free metanephrines for PPGL	Diagnostic accuracy, *n* = 858 (214 PPGL)	Low–some	Not serious	Not serious	Not serious	Undetected	Se 99%; Sp 89%	Moderate–high	(94)
7	^68^Ga-DOTA-SSTR PET detection (PPGL)	Diagnostic accuracy MA	Low–some	Not serious	Not serious	Not serious	Undetected	Per-lesion 92%–100%	Moderate–high	(95)
8	PA excess CV risk vs. essential HTN	Observational MA, 31 studies, 3,838 PA/9,284 EH	Moderate	Some	Not serious	Not serious	Suspected	Stroke OR 2.58; AF 3.52; CAD 1.77; HF 2.05	Moderate	(63)
9	Second-generation RDN vs. sham (ambulatory SBP)	SPYRAL OFF/ON-MED; RADIANCE SOLO/TRIO/II; REQUIRE negative	Low–some	Serious (REQUIRE; HTN-3 first-gen)	Some (mixed off-med/on-med)	Some (modest 4–7 mm Hg)	Possible	Daytime/24-h SBP −3.9 to −7.4 mm Hg vs. sham (36-month ON-MED −10.0)	Moderate	(107–117)
10	PA testing among resistant HTN (implementation)	2 large US cohorts (VHA *n* = 269,010; Kaiser)	Moderate	Not serious (1.6% and 2.1%)	Not serious	Not serious	Undetected	Only 1.6%–2.1% of eligible patients tested	Moderate	(119, 120)

GRADE domains rated per outcome. “Some” denotes a minor concern not warranting a full downgrade.

**Table 6:** Guideline-concordance matrix.

Recommendation	ES 2016 (18)	ES 2025 (17)	AHA 2017 (14)	AHA 2025 (10)	ESC 2024 (11)	ESH 2023 (12)	Note
Screen ALL hypertension for PA	No—high-risk only	Suggest (weak)	No	No—targeted	No—targeted	No—targeted	Largest active discordance
Screen resistant HTN for PA	Yes	Yes	Yes	Yes	Yes	Yes	Universal agreement—yet only 1.6%–2.1% are tested (119, 120)
Screen resistant HTN for OSA	—	—	Yes	Yes	Yes	Yes	OSA is the most prevalent associated secondary phenotype (81)
MRA as preferred fourth drug (resistant HTN)	—	—	Yes	Yes	Yes	Yes	PATHWAY-2, GRADE High (104)
AVS before adrenalectomy (when available)	Yes (exceptions)	Individualise	Yes if surgery	Yes if surgery	Yes if surgery	Yes if surgery	SPARTACUS vs. PASO/MATCH tension (69, 72, 74)
Renin-guided MRA intensity in bilateral PA	Not standardised	Yes (PAMO-aligned)	Not specified	Emerging	Emerging	Emerging	PAMO 2025 is the outcome standard (75)
Routine stenting of atherosclerotic RAS	—	—	No	No	No	No	ASTRAL, CORAL, STAR, DRASTIC (88–91)
RDN as an option after work-up	—	—	No (post HTN-3)	Consider, selected	Consider, selected	Consider, selected	Second-generation sham RCTs + 2023 ESC consensus (25, 107–117)
Plasma-free metanephrines first-line for PPGL	—	—	Yes	Yes	Yes	Yes	(20, 94)
Cause-directed surgery for Cushing/unilateral PA/PPGL	Yes (PA)	Yes (PA)	Yes	Yes	Yes	Yes	Precision principle across aetiologies

ISH 2020 (13) and WHO 2021 (16) are global, resource-stratified documents that provide screening and pharmacological frameworks but do not independently operationalise universal (“screen-all”) PA screening. “—” denotes a recommendation outside the scope of that document.

**Table 7:** Clinically relevant subgroups.

Subgroup	Definition	Finding (verified)	References
Age <40 vs. ≥40 years	Young-onset hypertension	Enriches for monogenic disease, FMD, and PA	(23, 56–60, 92)
Resistant hypertension	BP above goal on ≥3 drugs including a diuretic, or controlled on ≥4	Enriches OSA (64%) and PA (11%–20%); MRA preferred fourth drug; only 1.6%–2.1% tested	(5, 38, 81, 104, 119, 120)
Sex	Female vs. male	FMD ∼91% female; *KCNJ5*-mutant APA predominates in younger women	(51, 52, 92)
Setting	Primary care vs. tertiary referral	PA 4%–6% newly diagnosed/primary care vs. 11% referral (PAPY) vs. 11%–20% in resistant clinics	(34, 35, 37, 39)
Ancestry/region	East Asia vs. Europe/US	Xu: PA ≥4% in newly diagnosed Chinese HTN; JPAS excess target-organ damage; REQUIRE a negative RDN outlier	(39, 65, 117)
Renin on MRA	Suppressed vs. unsuppressed during medical therapy of PA	Suppressed renin marks residual CV and renal risk (Hundemer); PAMO makes unsuppressed renin the target	(66, 67, 75)

**Table 8:** Evidence-gap map.

Aetiology	Mechanism	Diagnosis	Therapy	Implementation/equity
Primary aldosteronism	Strong	Strong	Moderate	Sparse
Renovascular (atherosclerotic)	Moderate	Moderate	Moderate	Moderate
Fibromuscular dysplasia	Sparse	Moderate	Sparse	Sparse
Obstructive sleep apnoea	Moderate	Strong	Moderate	Sparse
PPGL	Strong	Strong	Moderate	Sparse
Cushing's syndrome	Moderate	Moderate	Moderate	Sparse
Monogenic	Strong	Moderate	Sparse	Sparse
Renal denervation	Moderate	Emerging	Moderate	Sparse

Cell values are Strong/Moderate/Sparse/Emerging. “Sparse” implementation evidence for primary aldosteronism is quantitative (119, 120), not rhetorical.

**Figure 1:**
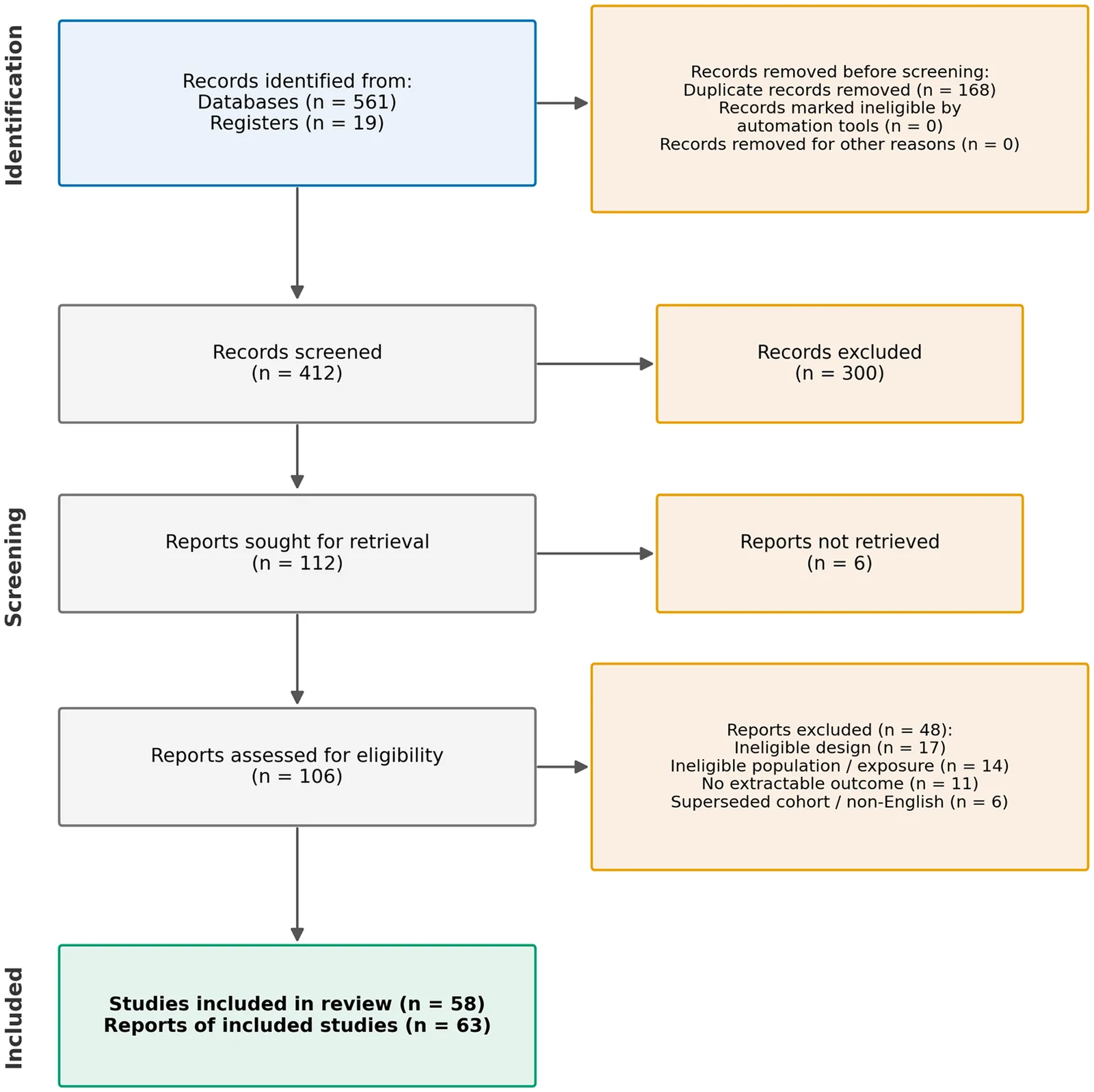
PRISMA 2020 flow of study selection. Formal search records progressed from 580 identified records (561 bibliographic database records and 19 registry records) to 412 records after removal of 168 duplicates; 112 full texts were sought, 106 were assessed, and 58 evidence records represented across 63 publications were included. The targeted post-search surveillance update through July 2026 is explicitly contextual and is not incorporated into these counts. PRISMA, Preferred Reporting Items for Systematic Reviews and Meta-Analyses. Source: formal review search record; reporting framework (26).

**Figure 2:**
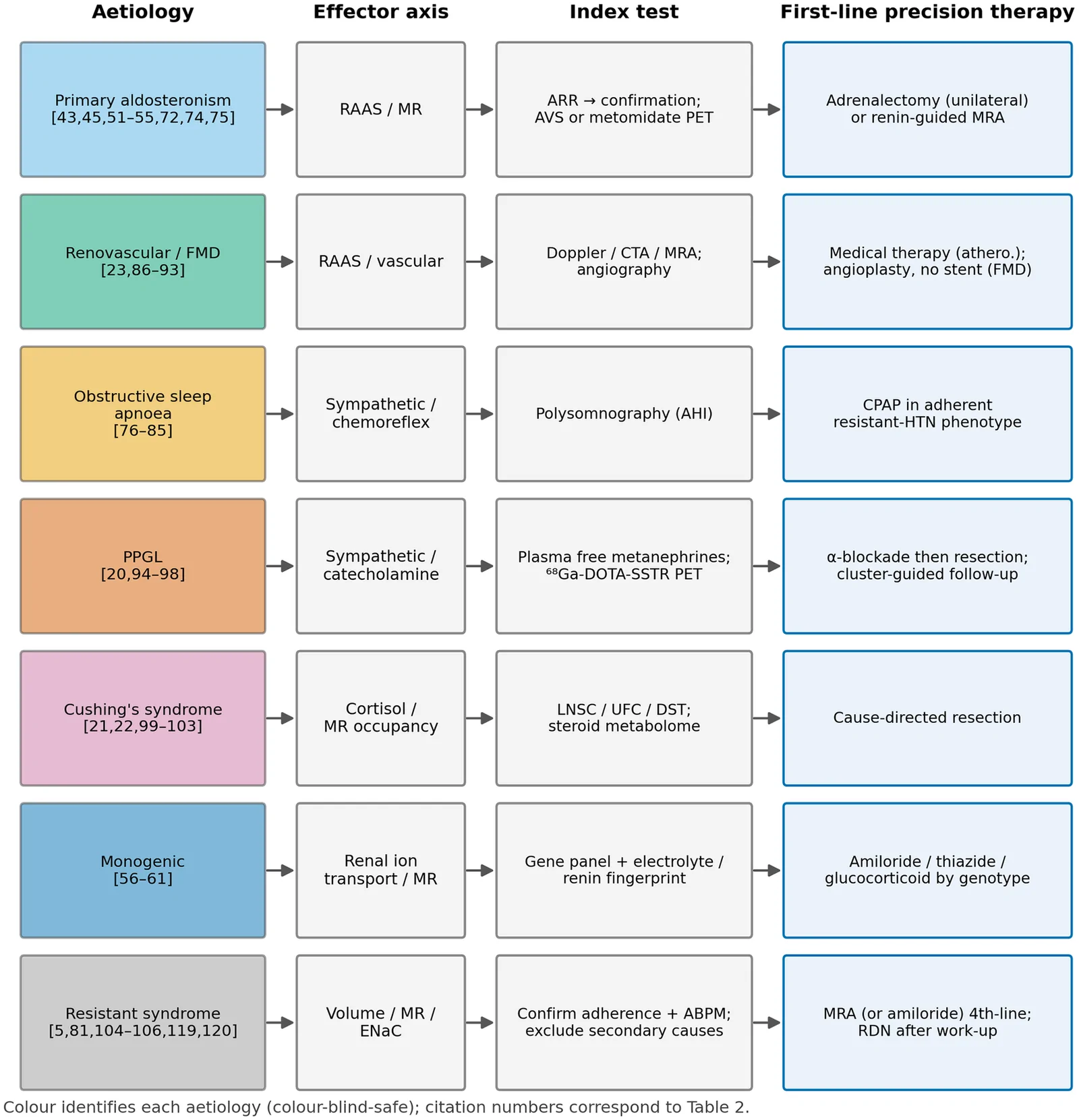
Mechanism → diagnostic test → precision therapy. Seven aetiology-specific pathways map the dominant effector axis to the index diagnostic test and first-line cause-directed treatment. RAAS, renin–angiotensin–aldosterone system; MR, mineralocorticoid receptor; ARR, aldosterone-to-renin ratio; AVS, adrenal vein sampling; PET, positron-emission tomography; FMD, fibromuscular dysplasia; CTA/MRA, computed tomography/magnetic resonance angiography; OSA, obstructive sleep apnoea; AHI, apnoea–hypopnoea index; CPAP, continuous positive airway pressure; PPGL, phaeochromocytoma/paraganglioma; LNSC, late-night salivary cortisol; UFC, urinary-free cortisol; DST, dexamethasone suppression test; ENaC, epithelial sodium channel; MRA, mineralocorticoid-receptor antagonist; RDN, renal denervation. Sources correspond to Table 2. Interpretation: causal distinctness determines how directly mechanism can be translated into a diagnostic test and a precision therapy.

**Figure 3:**
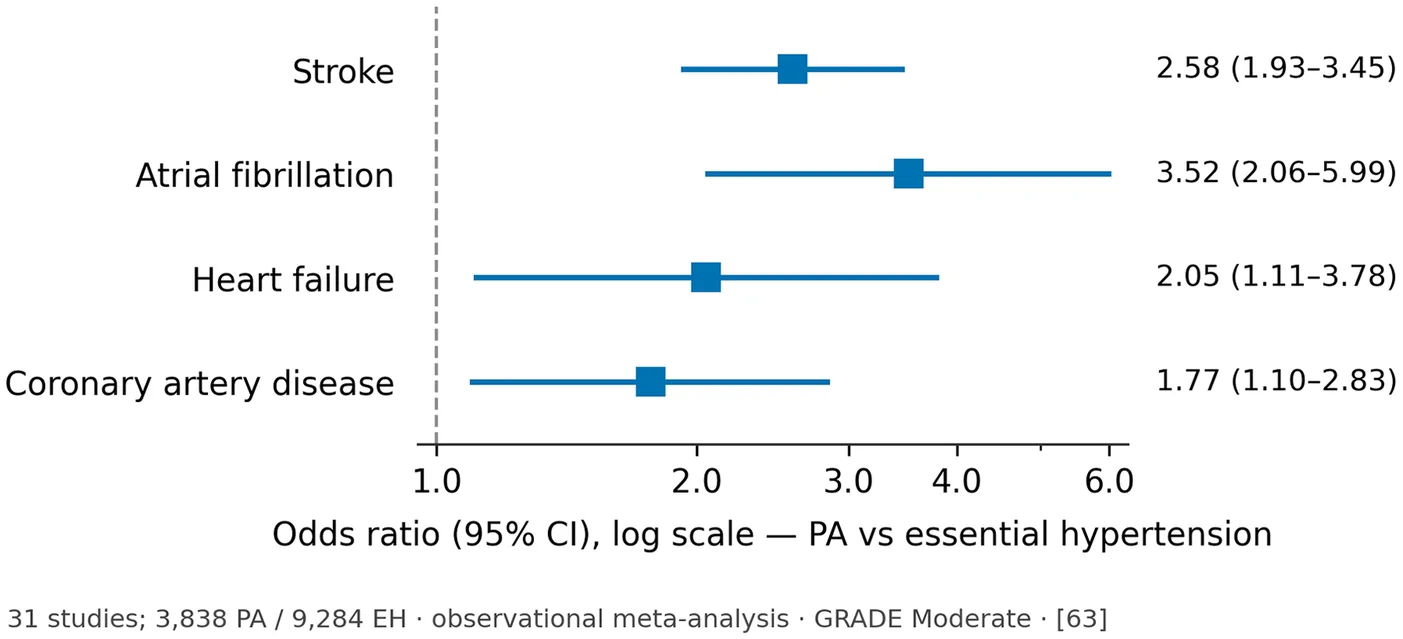
Cardiovascular outcomes in primary aldosteronism vs. essential hypertension. Study-level display of pooled odds ratios reported by Monticone et al. (63) from 31 studies (3,838 participants with PA and 9,284 with essential hypertension): stroke OR 2.58 (95% CI 1.93–3.45), atrial fibrillation OR 3.52 (2.06–5.99), heart failure OR 2.05 (1.11–3.78), and coronary artery disease OR 1.77 (1.10–2.83). The figure reproduces the published meta-analytic estimates; no re-pooling was performed in this review. PA, primary aldosteronism; OR, odds ratio; CI, confidence interval. Source: Monticone et al. (63).

**Figure 4:**
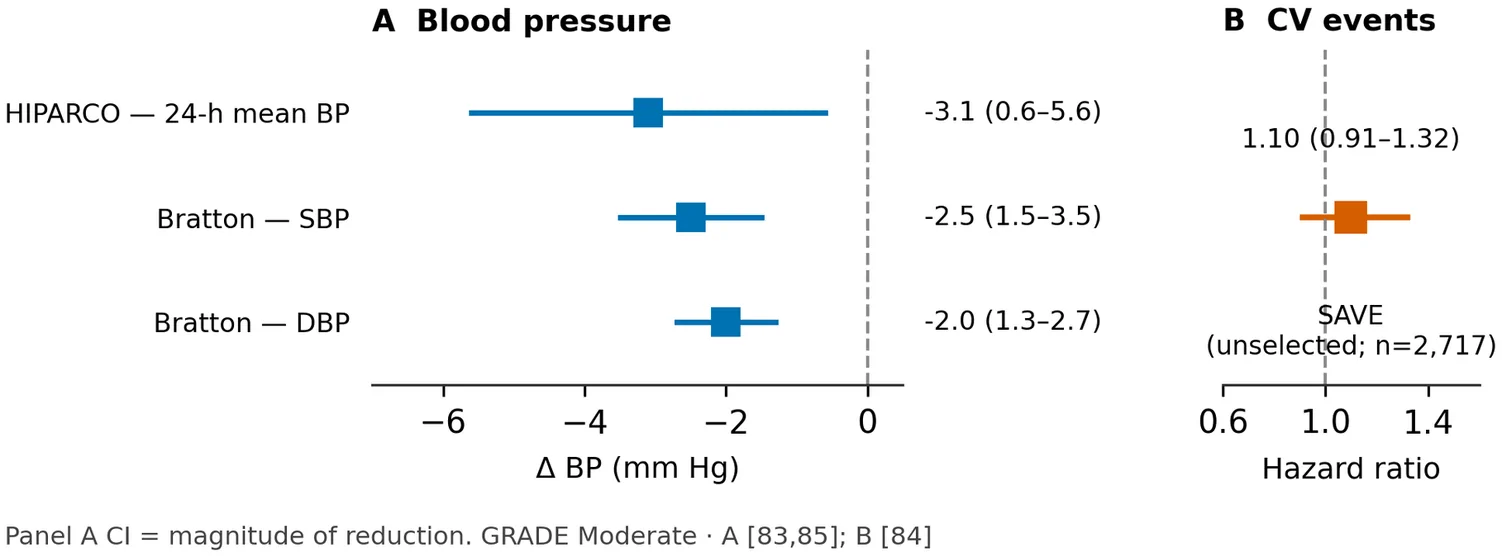
Continuous positive airway pressure: blood pressure and cardiovascular outcomes. **(A)** displays the HIPARCO 24 h mean BP reduction of 3.1 mm Hg (95% CI 0.6–5.6) in resistant hypertension and the Bratton meta-analysis estimates for SBP (−2.5 mm Hg, 95% CI 1.5–3.5 in magnitude) and DBP (−2.0 mm Hg, 1.3–2.7 in magnitude). **(B)** displays the SAVE cardiovascular HR 1.10 (95% CI 0.91–1.32). These are source-specific estimates and are not pooled together. CPAP, continuous positive airway pressure; BP, blood pressure; SBP, systolic BP; DBP, diastolic BP; HR, hazard ratio; CI, confidence interval. Sources: HIPARCO (83), SAVE (84), and Bratton et al. (85).

**Figure 5:**
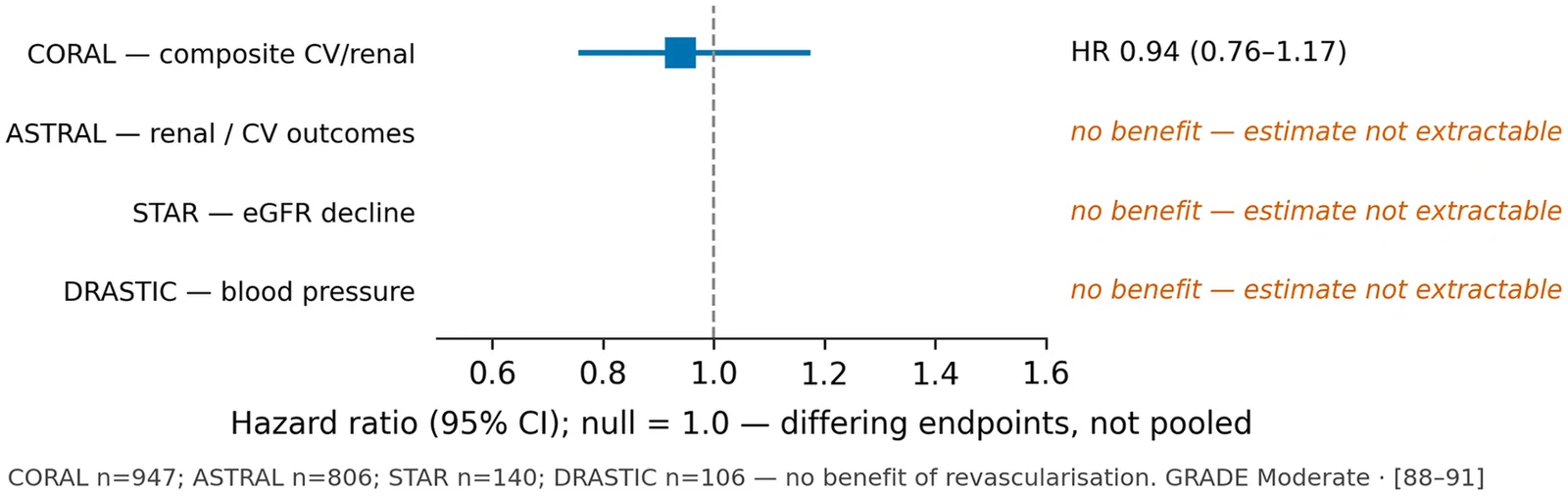
Atherosclerotic renal-artery revascularisation vs. medical therapy. Study-level display of randomised evidence. CORAL reported a cardiovascular/renal composite HR of 0.94 (95% CI 0.76–1.17); ASTRAL, STAR, and DRASTIC also failed to demonstrate routine clinical benefit. Because primary endpoints differ, no pooled summary estimate is calculated. HR, hazard ratio; CI, confidence interval; eGFR, estimated glomerular filtration rate. Sources: CORAL, ASTRAL, STAR, and DRASTIC (88–91).

**Figure 6:**
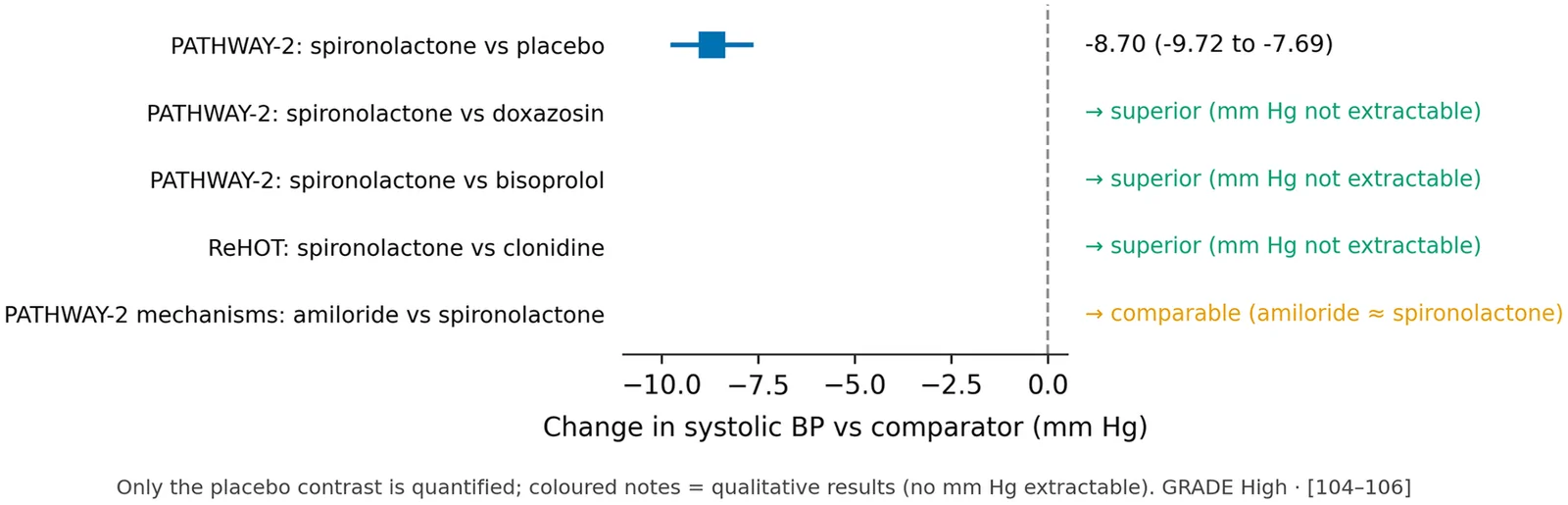
Fourth-line therapy in resistant hypertension. Study-level effect display anchored by PATHWAY-2: spironolactone reduced home SBP by 8.70 mm Hg vs. placebo (95% CI −9.72 to −7.69) and was superior to doxazosin and bisoprolol; ReHOT supported spironolactone over clonidine, while the PATHWAY-2 mechanistic substudy showed a comparable response to amiloride. Qualitative comparisons are labelled as such where a directly comparable millimetre-of-mercury estimate was not extractable; no pooled estimate is implied. SBP, systolic blood pressure; CI, confidence interval. Sources: PATHWAY-2, PATHWAY-2 mechanisms, and ReHOT (104–106).

**Figure 7:**
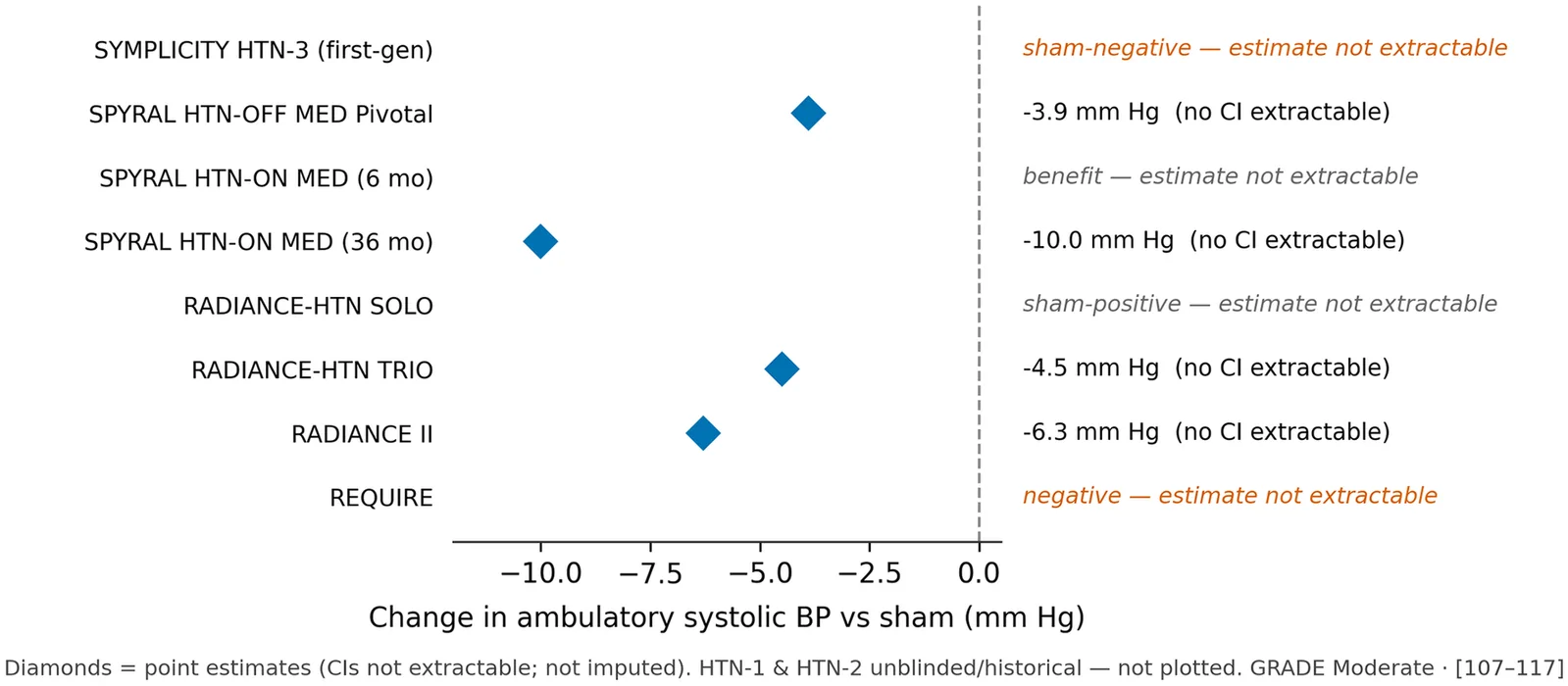
Catheter-based renal denervation vs. sham: ambulatory systolic blood pressure. Study-level display of sham-controlled trials. Quantitative point estimates include SPYRAL HTN-OFF-MED (−3.9 mm Hg), SPYRAL HTN-ON-MED at 36 months (−10.0 mm Hg), RADIANCE-HTN TRIO (−4.5 mm Hg) and RADIANCE II (−6.3 mm Hg); SYMPLICITY HTN-3 and REQUIRE were negative, while RADIANCE-HTN SOLO and SPYRAL HTN-ON-MED at 6 months were positive but are shown qualitatively where a single comparable estimate was not extracted. Confidence intervals are not imputed. This is not a pooled forest plot. RDN, renal denervation; SBP, systolic blood pressure; HTN, hypertension. Sources: (107–117).

**Figure 8:**
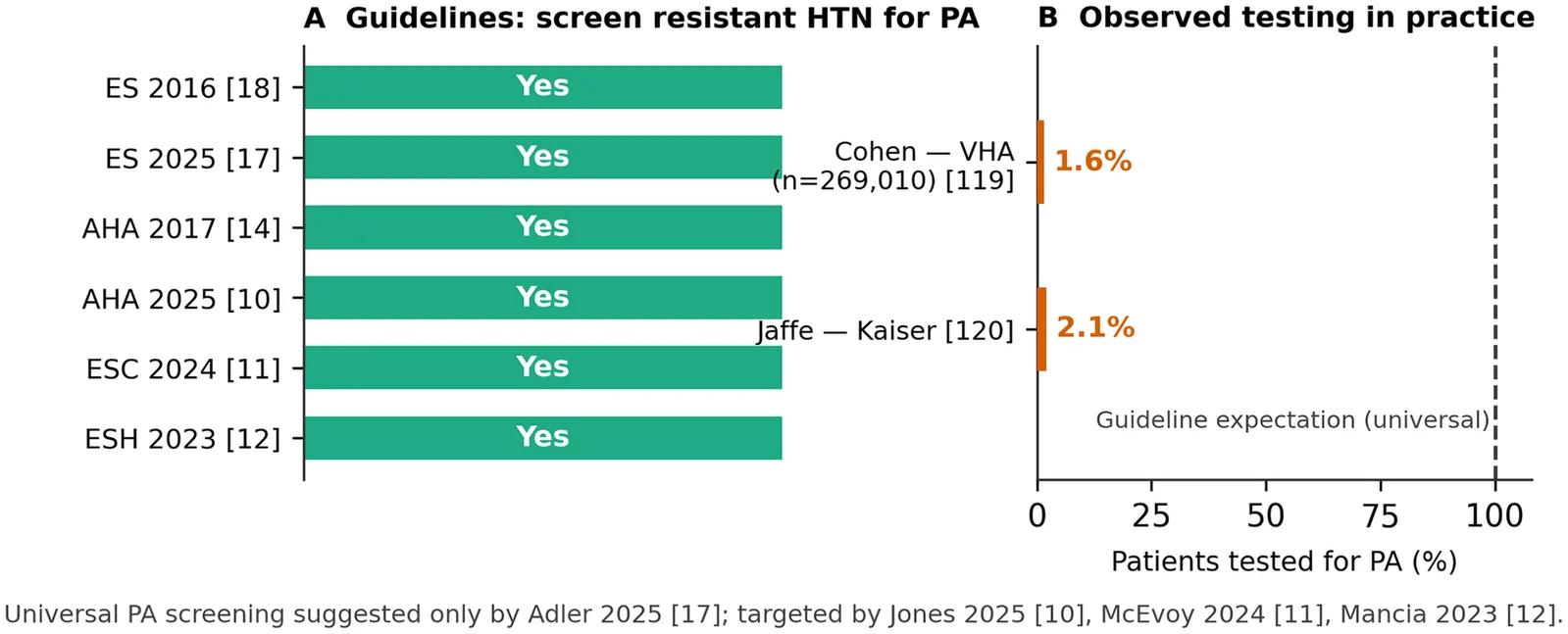
The implementation gap in primary aldosteronism screening. **(A)** shows concordant guideline endorsement of PA screening in resistant hypertension; **(B)** contrasts this with observed testing of only 1.6% in the US Veterans Health Administration cohort and 2.1% in an integrated-care cohort. The 100% reference line denotes the expectation that eligible resistant-hypertension patients should be screened; it does not imply that all hypertension guidelines recommend universal PA screening. PA, primary aldosteronism; HTN, hypertension; ES, endocrine society; AHA, American Heart Association; ESC, European Society of Cardiology; ESH, European Society of Hypertension; VHA, Veterans Health Administration. Sources: guideline documents (10–12, 14, 17, 18) and implementation cohorts (119, 120).

## 2 Methods

This systematic review was conducted and reported in accordance with the Preferred Reporting Items for Systematic Reviews and Meta-Analyses (PRISMA 2020) statement (26). Diagnostic accuracy components were reported with reference to PRISMA-DTA (27), while search reporting was additionally informed by PRISMA-S (121). The review was not prospectively registered with PROSPERO. Eligibility criteria, data-extraction domains, and synthesis categories were defined before the final evidence synthesis. Because review procedures were performed by a single reviewer (the first author), all study selection, data extraction, source verification, risk-of-bias appraisal, and certainty assessment were performed by the first author; independent duplicate screening, Cohen's kappa, and third-reviewer adjudication were therefore not applicable.

### 2.1 Eligibility criteria

Studies were eligible if they enrolled adults (aged ≥18 years) with hypertension and reported, for one or more secondary causes—primary aldosteronism, renovascular disease including fibromuscular dysplasia, obstructive sleep apnoea, phaeochromocytoma and paraganglioma, Cushing's syndrome or monogenic hypertension—data on at least one of the following: prevalence, disease mechanism, diagnostic test accuracy, or the effect of cause-directed treatment on blood pressure, biochemical, cardiovascular, or renal outcomes. Eligible designs comprised randomised controlled trials, controlled observational and cohort studies, cross-sectional prevalence studies, diagnostic accuracy studies, mechanistic studies, human genetic studies, and systematic reviews/meta-analyses if they provided an established pooled estimate relevant to the review question. Society guidelines and consensus statements were examined separately as normative reference standards for the guideline-concordance analysis and were not treated as primary therapeutic trials. Editorials, narrative reviews without extractable data, non-human studies, reports unavailable in English full text, and small non-monogenic case reports/series (<10 participants) without a unique diagnostic or therapeutic contribution were excluded. Monogenic kindreds were retained if they established a causal genotype–phenotype relationship.

### 2.2 Information sources

Five bibliographic databases—MEDLINE (via PubMed), Embase, the Cochrane Central Register of Controlled Trials (CENTRAL), Web of Science Core Collection and Scopus—and two trial registers—ClinicalTrials.gov and the WHO International Clinical Trials Registry Platform (ICTRP)—were searched from database inception to 30 June 2025. The electronic search was supplemented by backward and forward citation searching of included studies and relevant guidelines and by retrieval of guidance from the Endocrine Society, AHA/ACC, ESC, ESH, ISH, and WHO. No date or language restriction was applied at the electronic-search stage; English full-text availability was an eligibility criterion applied during screening.

### 2.3 Search strategy

The search combined controlled vocabulary (MeSH and Emtree) with free-text terms across three concept blocks. The first block captured hypertension (hypertension, hypertens*, high blood pressure); the second block captured secondary aetiologies (primary aldosteronism, hyperaldosteronism, aldosterone, renal-artery obstruction/stenosis, renovascular hypertension, fibromuscular dysplasia, obstructive sleep apnoea, pheochromocytoma/phaeochromocytoma, paraganglioma, Cushing syndrome, Liddle syndrome, pseudohypoaldosteronism type II/Gordon syndrome, glucocorticoid-remediable aldosteronism, and related monogenic hypertension terms); and the third block captured the review domains (prevalence, screening, diagnosis, sensitivity, specificity, adrenal vein sampling, molecular/genetic testing, treatment, mineralocorticoid-receptor antagonism, renal denervation, continuous positive airway pressure, adrenalectomy, and cause-directed therapy). Terms were combined with AND across concept blocks and OR within blocks and translated to the syntax of each database/register. The formal identification set comprised 561 bibliographic database records and 19 registry records (580 total), as reported in the PRISMA flow diagram. A targeted surveillance update through 31 July 2026 covered major society guidance and pivotal late-indexed/newly published phase three reports relevant to secondary or aldosterone-mediated hypertension. This update was used only for contextual interpretation and did not alter the original 580-record PRISMA flow, the 58-record formal synthesis, or the formal GRADE set.

Database-specific search strategies, including controlled-vocabulary and free-text syntax for MEDLINE/PubMed, Embase, CENTRAL, Web of Science, Scopus, ClinicalTrials.gov, and WHO ICTRP, are supplied in Supplementary Appendix 1. The verified formal record totals retained from the search are 561 bibliographic database records and 19 registry records (580 total). Source-specific hit counts for individual bibliographic databases were not retrospectively imputed where they were not separately preserved in the search record; the aggregate PRISMA counts therefore remain unchanged.

### 2.4 Selection process

Retrieved records were exported to a reference manager and deduplicated. The first author screened titles and abstracts against the prespecified eligibility criteria and then assessed potentially eligible reports in full text. Exclusion reasons were grouped as ineligible design, population/exposure, absence of an extractable outcome, overlapping/superseded report, unavailable full text, or other prespecified reasons. Because study selection was performed by a single reviewer, no inter-reviewer discrepancy resolution procedure or Cohen's *κ* statistic was applicable. The flow of records is shown in Figure 1; the numerical PRISMA flow was retained unchanged from the formal search record.

### 2.5 Data extraction

The first author extracted data using a standardised, structured workbook. Variables included the first author and publication year, study design, setting, sample size, aetiology, diagnostic index test and reference standard, intervention and comparator, duration of follow-up, serious adverse events, and effect estimates with 95% confidence intervals where available. DOI/PMID identifiers and study-level numerical values used in the manuscript, tables, and figures were checked against the corresponding published reports or bibliographic records during final reconciliation. The workbook in the Supplementary Material contains the full included-record extraction, mechanism mapping, diagnostic accuracy data, treatment outcomes, GRADE summary, subgroup analysis, evidence-gap map, guideline concordance, extractable effect estimates, and a separately labelled worksheet for the post-search contextual update.

### 2.6 Risk-of-bias and certainty assessment

Risk of bias was appraised with instruments matched to study design: the revised Cochrane RoB 2 tool for randomised trials (29), ROBINS-I for non-randomised intervention/observational evidence (30), QUADAS-2 for diagnostic accuracy studies (31), and AMSTAR 2 for systematic reviews/meta-analyses (32). Certainty of evidence for principal outcomes was summarised with GRADE (33), considering risk of bias, inconsistency, indirectness, imprecision, and publication bias. These ratings are single-reviewer, non-adjudicated structured judgments, and should not be interpreted as duplicate consensus ratings. Randomised evidence began at high certainty and could be downgraded, whereas observational evidence began at low certainty and could be upgraded for a large or dose-dependent effect when appropriate.

### 2.7 Synthesis methods

Because the evidence spanned heterogeneous aetiologies, study designs, diagnostic standards, interventions, and outcome definitions, a structured narrative synthesis with harmonised evidence tables was used. No new meta-analysis was undertaken. Published pooled estimates from eligible meta-analyses were reported with their original confidence intervals, whereas individual-trial estimates were displayed only at the study level. Figures 2–7 are therefore effect displays or mechanistic summaries rather than *de novo* pooled forest plots; no summary estimate was calculated across incompatible outcomes or study designs. Guideline concordance was mapped in Table 6, clinically relevant subgroups in Table 7, and evidence gaps in Table 8. The complete extraction workbook allows manuscript statements to be traced to individual evidence records.

## 3 Results

### 3.1 Study selection

The selection process is summarised in Figure 1. The database and register searches yielded 580 records; after 168 duplicates were removed, 412 records were screened, of which 300 were excluded at the title/abstract stage. Full-text reports were sought for 112 records; 106 were retrieved and assessed for eligibility, and 48 were excluded for prespecified reasons. Fifty-eight evidence records met the inclusion criteria and were represented across 63 publications. These included prevalence cohorts, diagnostic accuracy studies, randomised trials, controlled or observational cohorts, mechanistic/human genetic studies, and selected systematic reviews/meta-analyses that provided directly relevant pooled estimates. Contemporary society guidelines and consensus statements were examined separately for guideline concordance, while methodological, background, and post-search contextual references were included to complete the bibliography. The numerical PRISMA flow was not altered by the later contextual surveillance update.

### 3.2 Prevalence and the primary aldosteronism continuum

Primary aldosteronism is the most common surgically curable and specifically treatable and potentially surgically curable form of secondary hypertension, and its measured prevalence depends strongly on setting, biochemical thresholds, and testing method. In the prospective PAPY study of newly diagnosed referred hypertension, PA was identified in 11.2%, with aldosterone-producing adenoma in 4.8% and idiopathic hyperaldosteronism in 6.4% (34). In primary care, a prospective study found an overall prevalence of 5.9%, increasing from 3.9% in grade 1 to 11.8% in grade 3 hypertension (35). Brown et al. used sodium-modulated assessment of renin-independent aldosterone production and demonstrated a graded continuum: biochemically overt autonomous aldosterone production was present in 11.3% of normotensive participants, 15.7% of those with stage 1 hypertension, 21.6% of those with stage 2 hypertension, and 22.0% of those with resistant hypertension (36). These values should not be conflated with prevalence estimates obtained using conventional case definition in resistant-hypertension cohorts. Screening in practice rests on the aldosterone-to-renin ratio, whose performance varies with the assay, cut-off, posture, potassium status, and interfering drugs, and confirmation is commonly required before subtype assessment (17, 18). A meta-regression further showed that study methods and setting explain a substantial proportion of the apparent prevalence heterogeneity across studies (37).

In resistant hypertension, the yield is higher still. A retrospective series reported primary aldosteronism in 11.3% of resistant patients, with individual series ranging from roughly 5.5% to 20% (38); a prospective Chinese cohort of recently detected hypertension found a prevalence of at least 4% (39). Historical studies first signalled the scale of the problem: hyperaldosteronism was common among both Black and White subjects with resistant hypertension (40), and a multicentre report from five continents documented a marked increase in diagnosis, including surgically correctable forms, once systematic screening was applied (41). This resurgence of recognition was aptly characterised as a “renaissance” of the syndrome (42).

Contemporary physiology reframes primary aldosteronism as a continuum rather than a dichotomy. The expanding-spectrum synthesis by Vaidya et al. argued that renin-independent aldosterone production exists along a graded axis with implications for diagnosis, treatment, and research (43), and a subsequent update refined the diagnostic approach based on the same principle (44). Mechanistic studies situates the disorder both inside and outside the kidney (45); a normotensive continuum of renin-independent aldosteronism has been demonstrated (46); and a cohort study established that subclinical autonomous aldosterone production predicts incident hypertension (47). Clinical reviews synthesise this evidence for practice (48, 49), and the European Society of Hypertension working group has issued a position statement on the genetics, prevalence, screening, and confirmation of the disorder (50). The practical significance of the continuum model is that the binary question—does this patient have primary aldosteronism, yes or no—is replaced by a graded one about the degree of renin-independent aldosterone production and its cardiovascular consequences. That reframing helps resolve much of the historical dispute about prevalence, because different thresholds applied to a single continuous distribution necessarily yield different counts, and it shifts the clinical emphasis from diagnostic labelling towards quantifying and treating an injurious hormonal axis when identified. The mechanistic architecture underlying these observations is set out in Table 2.

### 3.3 Mechanisms from cell to blood pressure

The molecular drivers of aldosterone-producing adenomas are now well defined. Somatic mutations in the potassium channel gene *KCNJ5* were the first recurrent drivers to be identified (51), and characterisation of the wider genetic spectrum of aldosterone-producing adenomas established their clinical correlates (52). Somatic and germline mutations in the calcium-channel gene *CACNA1D* (53) and somatic mutations in the cation transporter genes *ATP1A1* and *ATP2B3* (54) complete the principal somatic landscape, each converging on increased intracellular zona glomerulosa calcium signalling and constitutive *CYP11B2*-driven aldosterone synthesis. With age, aldosterone-producing cell clusters accumulate and can drive autonomous secretion, blurring the boundary between disease and ageing (55).

The Mendelian forms of hypertension illuminate the same effector pathways through rare, high-penetrance lesions. Glucocorticoid-remediable aldosteronism arises from a chimaeric *CYP11B1/CYP11B2* gene placing aldosterone synthase under adrenocorticotropic hormone (ACTH) control (56). Liddle's syndrome results from gain-of-function mutations in the epithelial sodium channel (57). Pseudohypoaldosteronism type II (Gordon's syndrome) is caused by mutations in the WNK kinases (58) and in their regulators, kelch-like 3 and cullin 3 (59), whereas an activating mineralocorticoid-receptor mutation causes hypertension that is exacerbated by pregnancy (60). These monogenic disorders map directly to renal sodium handling and mineralocorticoid-receptor signalling and, importantly, to specific therapies. The therapeutic corollary is exact: epithelial sodium channel blockade with amiloride controls Liddle's syndrome, in which a mineralocorticoid-receptor antagonist would fail, a thiazide diuretic corrects the sodium-chloride cotransporter overactivity of Gordon's syndrome, and low-dose glucocorticoid therapy suppresses the ACTH-driven chimeric aldosterone synthase of glucocorticoid-remediable aldosteronism, so that genotype, rather than blood pressure alone, dictates drug. Beyond the monogenic disorders, the broader genetic architecture of blood pressure is polygenic, with molecular and genome-wide analyses identifying numerous common variants of small effect (61, 62). The full mechanism-to-therapy mapping is shown in Figure 2.

### 3.4 Cardiovascular and renal harm in primary aldosteronism, and surgical and medical outcomes

Aldosterone excess is not merely a marker of higher blood pressure; it causes mineralocorticoid-receptor–mediated cardiovascular and renal injury out of proportion to the level of blood pressure. The pivotal meta-analysis by Monticone et al., which compared primary aldosteronism with essential hypertension across 31 studies (3,838 patients with primary aldosteronism and 9,284 with essential hypertension), found substantially higher odds of stroke [odds ratio 2.58, 95% confidence interval (CI) 1.93–3.45], atrial fibrillation (3.52, 2.06–5.99), heart failure (2.05, 1.11–3.78), and coronary artery disease (1.77, 1.10–2.83) (63). These estimates are displayed in Figure 3. Earlier and subsequent cohorts are concordant: an initial report documented an increased rate of cardiovascular events in primary aldosteronism (64), and the multicentre Japanese JPAS study confirmed excess cardiovascular disease and target-organ damage (65). Critically, this excess risk is modifiable but conditional on effective mineralocorticoid-receptor blockade: in a retrospective cohort of medically treated patients, those whose renin remained suppressed during treatment with a mineralocorticoid-receptor antagonist retained excess cardiometabolic risk and mortality relative to essential hypertension, whereas those with unsuppressed renin did not (66). Renal outcomes follow the same logic, with medically and surgically treated patients showing distinct trajectories of kidney function (67). Taken together, these cohorts reframe the therapeutic target in medically managed disease from blood pressure alone to biochemical control, with unsuppressed renin serving as a pragmatic bedside signal that mineralocorticoid-receptor blockade is adequate and that the receptor-mediated component of cardiovascular and renal risk has been addressed. Mass spectrometry–based steroid profiling adds mechanistic and subtyping information, including recognition of glucocorticoid cosecretion (68).

Subtyping determines whether disease is surgically curable, and the evidence base is nuanced. The SPARTACUS randomised trial found no 1-year difference in antihypertensive drug intensity between computed tomography–guided and adrenal vein sampling–guided strategies (69). Nevertheless, adrenal vein sampling remains the reference standard for lateralisation where expertise exists; the AVIS study showed centre-dependent technical success with a low complication rate of approximately 0.61% (70), and expert consensus codifies its use (71). In the MATCH within-patient trial (*n*=143), [^11^C]metomidate PET–CT performed comparably to adrenal vein sampling, with surgical success in 77 of 78 operated patients (72), building on earlier tracer-development work (73). The PASO international cohort then established standardised postoperative outcomes in 705 patients across 12 centres: complete clinical success was achieved in 259/705 (37%; centre range 17%–62%), partial clinical success in 334/705 (47%), and complete biochemical success in 656/699 (94%; centre range 83%–100%) (74). This separation is clinically important: biochemical cure is common after correctly selected unilateral adrenalectomy, whereas complete freedom from hypertension is less frequent because vascular and renal consequences of longstanding hypertension may persist. The medical-treatment counterpart, PAMO (*n* = 1,258), identified unsuppressed renin during mineralocorticoid-receptor antagonist therapy as a biochemical marker of adequate treatment (75). The diagnostic, therapeutic, and certainty evidence for PA is summarised in Tables 3–5.

### 3.5 Obstructive sleep apnoea

Obstructive sleep apnoea is common and frequently undiagnosed, with a literature-based analysis estimating that close to a billion adults worldwide have the condition at least mildly (76). Its link to hypertension is mechanistically direct. Foundational human work showed that obstructive apnoeas provoke marked sympathetic activation, recorded as increased muscle sympathetic nerve activity, that persists into wakefulness (77), and an American Heart Association statement synthesised the cardiovascular consequences (78). Longitudinal cohorts established causality for incident hypertension: the Wisconsin Sleep Cohort demonstrated a dose-dependent association between sleep-disordered breathing and the development of hypertension (79), corroborated cross-sectionally by the Sleep Heart Health Study (80). In resistant hypertension, the disorder is the single most prevalent associated secondary condition: a prospective study found obstructive sleep apnoea in 64% of patients with resistant hypertension, the most common secondary phenotype in that population (81), and unrecognised obstructive sleep apnoea was present in up to 83% of patients with drug-resistant hypertension (82).

Treatment effect, however, is phenotype dependent. In the HIPARCO randomised trial of patients with obstructive sleep apnoea and resistant hypertension, continuous positive airway pressure reduced 24-h mean blood pressure by 3.1 mm Hg (95% CI 0.6–5.6) and improved the nocturnal dipping pattern (83). The restoration of dipping is mechanistically coherent, since the recurrent nocturnal sympathetic surges of untreated apnoea are precisely what abolish the normal night-time fall in pressure, and their abolition is the proximate route to lower ambulatory readings. By contrast, in the large SAVE trial of patients with obstructive sleep apnoea and established cardiovascular disease, continuous positive airway pressure did not reduce cardiovascular events (hazard ratio 1.10, 95% CI 0.91–1.32), in the context of modest adherence averaging 3.3 h per night (84). A meta-analysis quantified the class-level blood-pressure effect of positive airway pressure and mandibular advancement devices (85). The blood pressure and event data are shown in Figure 4. The message is one of selection: benefit on blood pressure is realised in adherent patients with the resistant-hypertension phenotype, whereas unselected event prevention is not demonstrated.

### 3.6 Renovascular hypertension and fibromuscular dysplasia

Renovascular hypertension arises when renal hypoperfusion activates the renin–angiotensin–aldosterone system; the pathophysiology and clinical evaluation of renal-artery stenosis are set out in standard reviews (86, 87). The decisive therapeutic question—whether to revascularise atherosclerotic disease—has been answered consistently by randomised trials, and the answer is that routine stenting should not be performed. In CORAL (*n* = 947), stenting added to medical therapy did not reduce the composite of cardiovascular and renal events (hazard ratio 0.94, 95% CI 0.76–1.17) (88). ASTRAL (*n* = 806) found no benefit of revascularisation on renal or cardiovascular outcomes (89), STAR (*n* = 140) showed no benefit on the decline in renal function (90), and the earlier DRASTIC trial (*n* = 106) found no durable blood-pressure advantage from angioplasty over medical therapy (91). These trials are displayed together in Figure 5, with the caution that their differing primary endpoints preclude a single pooled summary. The consistent lesson is that anatomical stenosis is not equivalent to physiological significance, and intervention should be reserved for carefully selected physiology, such as rapidly deteriorating function or flash pulmonary oedema. In these selected physiological circumstances, the balance of the randomised evidence permits intervention, but the default position for stable atherosclerotic disease is medical therapy with aggressive risk-factor modification.

Fibromuscular dysplasia is a distinct, non-atherosclerotic arteriopathy with a strong female predominance: the United States registry found that approximately 91% of patients were female (92). An American Heart Association scientific statement reviewed its biology and unanswered questions (93), and the 2019 international consensus provides current diagnostic and management standards, including angioplasty without stenting for focal renal disease (23).

### 3.7 Phaeochromocytoma, paraganglioma, and Cushing's syndrome

For catecholamine-secreting tumours, biochemical testing is highly accurate. In the reference diagnostic accuracy study, plasma-free metanephrines showed a sensitivity of 99% (211/214) and a specificity of 89% (586/644), establishing them as the first-line test (94). Functional imaging with ^68^Ga-DOTA-conjugated somatostatin receptor PET achieves high per-lesion detection, between 92% and 100% in meta-analysis, and is superior in SDHx-related and metastatic disease (95). The clinical spectrum and cluster biology of phaeochromocytoma and paraganglioma—pseudohypoxic cluster 1 (SDHx, VHL, EPAS1) vs. kinase-signalling cluster 2 (RET, NF1, MAX, TMEM127)—are reviewed comprehensively (96, 97), and recommendations from the First International Symposium codify perioperative *α*-blockade and follow-up (98). Germline status, particularly SDHB, intensifies lifelong surveillance for metastatic and multifocal disease. In practice, biochemical confirmation with plasma-free metanephrines precedes anatomical and functional imaging, and germline testing then stratifies both the choice of functional tracer and the intensity of follow-up, so that a single molecular result can inform diagnosis, localisation, and surveillance at once.

In Cushing's syndrome, cortisol excess raises blood pressure partly through saturation of renal 11*β*-hydroxysteroid dehydrogenase type 2, permitting illicit occupation of the mineralocorticoid receptor by cortisol; the disorder is reviewed in successive syntheses (99, 100). Plasma steroid metabolome profiling assists diagnosis and subtyping (101). Consensus guidance addresses the diagnosis and management of pituitary Cushing's disease (102), and the European Society of Endocrinology guideline governs the work-up of the adrenal incidentalomas that frequently prompt evaluation (103). The Endocrine Society documents on the diagnosis (21) and treatment (22) of Cushing's syndrome frame cause-directed resection as first-line therapy in expert centres. The unifying mechanism across these adrenal and pituitary disorders is that a discrete hormonal excess drives the hypertension, so that removing or blocking the source, rather than escalating non-specific antihypertensives, is both the rational and the definitive treatment.

### 3.8 Resistant hypertension therapy and renal denervation

For resistant hypertension, the pharmacological evidence is unusually strong. In the PATHWAY-2 crossover trial (*n* = 314), spironolactone was superior to placebo and to the active comparators bisoprolol and doxazosin, reducing home systolic blood pressure (SBP) by 8.70 mm Hg vs. placebo (95% CI −9.72 to −7.69) (104). The mechanistic substudies showed that resistant hypertension is characteristically a low-renin, volume-expanded state and that amiloride produced blood pressure reductions comparable to spironolactone, implicating epithelial sodium channel and mineralocorticoid receptor–dependent sodium retention (105). The ReHOT trial independently confirmed the superiority of spironolactone over clonidine as a fourth-line agent (106). This effect anchors the highest-certainty recommendation in the field and is displayed in Figure 6, with GRADE certainty rated High in Table 5.

Catheter-based renal denervation has a more turbulent evidence history that illustrates the value of sham control. The pivotal SYMPLICITY HTN-3 trial (*n* = 535) found no significant difference in office systolic blood pressure vs. a sham procedure (107), overturning the large reductions reported earlier by the unblinded HTN-2 trial (108) and the first-in-human cohort (109), whose open-label designs had overestimated the true effect. A second generation of properly sham-controlled trials then established a smaller but real effect. SPYRAL HTN-ON-MED demonstrated benefit at 6 months in the presence of antihypertensive drugs (110); SPYRAL HTN-OFF-MED Pivotal reduced 24-h systolic blood pressure by 3.9 mm Hg vs. sham off medication (111); and SPYRAL HTN-ON-MED reported a 10.0 mm Hg reduction at 36 months (112). The ultrasound programme was concordant: RADIANCE-HTN SOLO (113), RADIANCE-HTN TRIO (−4.5 mm Hg) (114), and RADIANCE II (daytime ambulatory systolic blood pressure −6.3 mm Hg) (115) were each sham-positive, and the open-label DENERHTN trial supported an additive effect within a standardised stepped-care regimen (116). The picture is not uniform, however: the REQUIRE trial in Japan and Korea was negative (117). These sham-controlled results are shown in Figure 7, with the first-in-human (109) and HTN-2 (108) studies annotated as unblinded and historical rather than plotted on the sham forest. Reflecting this balance, the ESC Council on Hypertension and EAPCI consensus positions denervation as an option for selected patients at experienced centres after thorough work-up (25). Patient selection and shared decision-making are therefore central: the honest counselling position is that denervation offers a durable but modest average reduction in blood pressure, of the order of a few millimetres of mercury on ambulatory measurement, that it is not a substitute for excluding a specific secondary cause, and that the response varies appreciably between individuals. For context, the SPRINT trial established the cardiovascular benefit of intensive blood-pressure control in high-risk essential hypertension, a separate question from secondary-cause management (118).

#### 3.8.1 Aldosterone synthase inhibition: post-search phase 3 evidence

Targeted post-search surveillance identified two phase 3 trials that strengthen the mechanistic relevance of aldosterone dysregulation in difficult-to-control hypertension but were not incorporated into the original PRISMA or GRADE cohort. In BaxHTN, 796 participants were randomised and 794 received baxdrostat 1 mg, baxdrostat 2 mg, or placebo. At 12 weeks, placebo-corrected seated SBP differences were −8.7 mm Hg (95% CI −11.5 to −5.8) with 1 mg and −9.8 mm Hg (−12.6 to −7.0) with 2 mg; serum potassium >6.0 mmol/L occurred in 2.3%, 3.0%, and 0.4%, respectively (122). In Launch-HTN (*n* = 1,083), pooled lorundrostat 50 mg reduced automated office SBP at week 6 by an additional 9.1 mm Hg vs. placebo (95% CI −13.3 to −4.9), with hyponatraemia, hyperkalaemia, and reductions in kidney function occurring more often with active treatment (123). These trials establish clinically meaningful blood-pressure lowering on background therapy, but they do not establish class-wide cardiovascular-outcome efficacy; electrolyte and renal-function monitoring, durability of effect, and long-term outcome data remain important evidence gaps.

### 3.9 Guideline concordance and the implementation gap

Guideline positions are catalogued in Table 6. The largest active discordance concerns the breadth of PA screening: the 2025 Endocrine Society guideline suggests screening all individuals with hypertension (17), whereas the 2025 AHA/ACC, 2024 ESC and 2023 ESH guidelines retain targeted case detection (10–12). For resistant hypertension, however, the major guidelines are concordant that PA should be sought. This agreement makes the implementation deficit particularly important: in 269,010 US veterans with apparent treatment-resistant hypertension, only 1.6% were tested for PA (119), and the corresponding proportion in an integrated health system was 2.1% (120). The evidence–practice gap is depicted in Figure 8. Clinically relevant subgroups are summarised in Table 7, and the remaining evidence gaps are presented in Table 8.

## 4 Discussion

The most consequential finding of this map is a matter of proportion. The discordance that attracts the most attention—whether to screen all hypertension for primary aldosteronism, as one 2025 Endocrine Society document suggests (17), or to screen selectively, as cardiology and hypertension guidelines advise (10–12)—is a difference in emphasis at the margin. It is small compared with the discordance between every guideline and actual practice: although screening resistant hypertension for primary aldosteronism is universally recommended, only 1.6%–2.1% of eligible patients are tested (119, 120). Debating the denominator for screening is of limited value, while the numerator of those actually tested remains near zero. The rate-limiting step to precision care in secondary hypertension is not the wording of guidelines but the act of testing. This reframing has a clear operational reading. The greatest marginal gain available to the field is not another refinement of a screening threshold but a reliable system-level mechanism—embedded prompts, standing order sets, reflex laboratory testing—that converts an eligible resistant-hypertension patient into a tested one. A recommendation is followed that 2% of the time functions, at the population level, almost as if it did not exist.

The therapeutic corollary is that precision in this field is fundamentally aetiology-specific, and the evidence supports differentiated actions rather than a single escalation pathway. In PA, subtype-selected adrenalectomy produces complete biochemical success in 94% of evaluable patients but complete clinical success in only 37%, underscoring the need to distinguish hormonal cure from cure of hypertension (74). For bilateral or medically managed PA, mineralocorticoid-receptor antagonist therapy should achieve biochemical as well as pressure control, with unsuppressed renin serving as a pragmatic treatment marker (66, 75). In stable atherosclerotic renal-artery stenosis, routine stenting should not be performed because four randomised trials fail to show a consistent clinical advantage (88–91). In obstructive sleep apnoea, continuous positive airway pressure produces a modest but clinically coherent blood pressure benefit in adherent resistant-hypertension phenotypes (83) but has not reduced cardiovascular events in unselected disease (84). Renal denervation produces a modest average sham-corrected reduction in ambulatory pressure and is an option only after appropriate secondary-cause work-up and shared decision-making (25, 107–117). The monogenic disorders provide the clearest proof of the precision principle because genotype can identify the appropriate drug class directly: amiloride for Liddle syndrome, thiazide for Gordon syndrome, and low-dose glucocorticoid for glucocorticoid-remediable aldosteronism (56–59).

The post-search phase 3 aldosterone synthase inhibitor data reinforce the centrality of aldosterone biology but should be interpreted within their evidentiary limits. BaxHTN and Launch-HTN each demonstrated an approximately 9 mm Hg placebo-corrected reduction in office systolic pressure on background therapy (122, 123). These are important blood pressure efficacy signals, not evidence of reduced myocardial infarction, stroke, heart failure, kidney failure, or mortality. Hyperkalaemia, hyponatraemia, and changes in kidney function also make laboratory monitoring part of the therapeutic proposition. Accordingly, this review does not infer class-wide cardiovascular benefit from short-term surrogate outcomes, and long-term comparative and outcome trials remain necessary.

Underpinning every one of these differentiated actions is a diagnostic step of defined accuracy, and it is worth emphasising how favourable that accuracy now is. Plasma-free metanephrines effectively exclude phaeochromocytoma when negative (94); somatostatin receptor PET localises disease that older imaging missed (95); the aldosterone-to-renin ratio, for all its pre-analytical fragility, identifies the large reservoir of unrecognised aldosteronism (36); and non-invasive [^11^C]metomidate PET–CT now approaches adrenal vein sampling for surgical decision-making (72). The tools required to convert mechanism into management already exist and perform well; the deficit is in their use, not their validity.

Implementation and equity remain the least mature evidence domain across most aetiologies (Table 8). This should be read primarily as a limitation of the field rather than a weakness unique to this review: diagnostic accuracy and treatment efficacy have often been established in specialist settings, whereas comparatively few studies examine how to deliver screening, confirmatory diagnostics and definitive therapy equitably across resource levels, geographical regions, and health systems. The striking 1.6%–2.1% PA testing rates in otherwise well-characterised resistant-hypertension populations illustrate how implementation failure can overwhelm even strong diagnostic and therapeutic evidence (119, 120).

Several limitations should be acknowledged. First, the review was not prospectively registered in PROSPERO. Second, it was conducted by a single author; the absence of independent duplicate screening, extraction and appraisal increases the possibility of selection, transcription, and judgement error despite the use of prespecified criteria and source-level reconciliation. Third, eligibility required an obtainable full-text English version, which can introduce language bias. Fourth, the formal search ended on 30 June 2025; a targeted surveillance update through July 2026 was therefore added to contextualise major late-indexed/new guidance and phase 3 trials, but those reports were deliberately kept outside the original PRISMA counts and formal GRADE cohort. Fifth, the review spans heterogeneous aetiologies, study designs, and outcomes, so no new quantitative pooling was attempted; published meta-analytic estimates and individual-trial effects are presented separately. Finally, several important domains remain dominated by observational evidence, including cardiovascular risk in PA and many implementation questions. The renal denervation literature remains heterogeneous, with negative trials such as SYMPLICITY HTN-3 and REQUIRE alongside otherwise positive second-generation sham-controlled studies.

A further reporting limitation is that the verified aggregate identification totals were preserved, whereas individual database-specific hit counts were not retained separately in the final search record. These counts have not been retrospectively reconstructed or allocated across databases, because doing so would risk introducing unsupported precision. The complete database-specific search syntax is nevertheless supplied to permit readers to judge scope and facilitate future replication.

These limitations define the research agenda. Future updates should repeat the full multi-database search and incorporate late-phase 3 evidence into a refreshed PRISMA cohort rather than relying on contextual surveillance. High-priority trials include cardiovascular and renal outcome studies of aldosterone synthase inhibitors; pragmatic implementation trials designed to increase PA testing in resistant and young-onset hypertension; randomised comparisons of renin-guided vs. conventional mineralocorticoid-receptor antagonist titration; physiology-based selection strategies for renovascular intervention; and equity-focused studies that test whether precision diagnostic pathways remain effective outside specialist centres.

## 5 Conclusions

Secondary hypertension is sufficiently common, especially in resistant and young-onset phenotypes, that under-recognition has population-level consequences. Across PA, renovascular disease, obstructive sleep apnoea, PPGL, Cushing's syndrome, and monogenic hypertension, pathophysiological mechanisms increasingly map to specific diagnostic tests and cause-directed treatments. For unilateral PA, biochemical cure after adrenalectomy is far more frequent than complete clinical cure, and this distinction should inform counselling. For resistant hypertension, spironolactone remains the evidence-based fourth-line standard, while renal denervation is reserved for selected patients after complete work-up. Emerging aldosterone synthase inhibitors provide substantial additional blood pressure lowering but do not yet have a proven cardiovascular-outcome benefit. The major translational opportunity is therefore not simply the discovery of new pathways, but the reliable identification of eligible patients and the delivery of the right diagnostic and therapeutic pathway at the right time.
