# Supplementary material for: Mechanism-defined secondary hypertension enables precision diagnosis and treatment: an evidence review
Source: Front Cardiovasc Med. 2026 Oct 14;13:1958980. doi: 10.3389/fcvm.2026.1958980 (PMC13714811; doi:10.3389/fcvm.2026.1958980)
Supplement: Supplementary file 1 [file Supplementaryfile1.docx]

Supplementary Appendix 1. Database-specific search strategies

Review: Secondary hypertension: mechanisms, precision diagnosis, and cause-directed therapy — a systematic review

Search coverage and counting note. The formal electronic search covered database inception to 30 June 2025. No date or language restriction was applied at the electronic-search stage; English full-text availability was applied during eligibility assessment. The verified formal identification totals are 561 bibliographic-database records and 19 trial-register records (580 total). The source-level hit counts for individual bibliographic databases were not separately retained in the final search record and have therefore not been retrospectively reconstructed or allocated. The database-specific syntax below documents the prespecified concept-block strategy and its source-specific translation. A targeted surveillance update through 31 July 2026 was used only for contextual interpretation and did not alter the formal PRISMA flow.

# MEDLINE via PubMed

("Hypertension"[Mesh] OR hypertens*[tiab] OR "high blood pressure"[tiab]) AND ("Hyperaldosteronism"[Mesh] OR "primary aldosteronism"[tiab] OR hyperaldosteronism[tiab] OR aldosterone[tiab] OR "Renal Artery Obstruction"[Mesh] OR "renal artery stenosis"[tiab] OR "renovascular hypertension"[tiab] OR "Fibromuscular Dysplasia"[Mesh] OR "Sleep Apnea, Obstructive"[Mesh] OR "obstructive sleep apnoea"[tiab] OR "obstructive sleep apnea"[tiab] OR pheochromocytoma[tiab] OR phaeochromocytoma[tiab] OR paraganglioma[tiab] OR "Cushing Syndrome"[Mesh] OR "Liddle syndrome"[tiab] OR "Gordon syndrome"[tiab] OR pseudohypoaldosteronism[tiab] OR "glucocorticoid-remediable aldosteronism"[tiab] OR "monogenic hypertension"[tiab]) AND (prevalence[tiab] OR screening[tiab] OR diagnosis[tiab] OR sensitivity[tiab] OR specificity[tiab] OR "adrenal vein sampling"[tiab] OR genetic*[tiab] OR molecular[tiab] OR treatment[tiab] OR "mineralocorticoid receptor antagonist"[tiab] OR spironolactone[tiab] OR amiloride[tiab] OR "renal denervation"[tiab] OR "continuous positive airway pressure"[tiab] OR adrenalectomy[tiab] OR therapy[tiab])

# Embase

('hypertension'/exp OR hypertens*:ti,ab,kw OR 'high blood pressure':ti,ab,kw) AND ('primary aldosteronism'/exp OR 'primary aldosteronism':ti,ab,kw OR hyperaldosteronism:ti,ab,kw OR aldosterone:ti,ab,kw OR 'renal artery stenosis'/exp OR 'renovascular hypertension':ti,ab,kw OR 'fibromuscular dysplasia'/exp OR 'obstructive sleep apnea'/exp OR pheochromocytoma/exp OR paraganglioma/exp OR 'cushing syndrome'/exp OR 'liddle syndrome':ti,ab,kw OR 'gordon syndrome':ti,ab,kw OR pseudohypoaldosteronism:ti,ab,kw OR 'glucocorticoid remediable aldosteronism':ti,ab,kw OR 'monogenic hypertension':ti,ab,kw) AND (prevalence:ti,ab,kw OR screening:ti,ab,kw OR diagnosis:ti,ab,kw OR sensitivity:ti,ab,kw OR specificity:ti,ab,kw OR 'adrenal vein sampling':ti,ab,kw OR genetic*:ti,ab,kw OR molecular:ti,ab,kw OR treatment:ti,ab,kw OR 'mineralocorticoid receptor antagonist':ti,ab,kw OR spironolactone:ti,ab,kw OR amiloride:ti,ab,kw OR 'renal denervation':ti,ab,kw OR 'continuous positive airway pressure':ti,ab,kw OR adrenalectomy:ti,ab,kw)

# CENTRAL

(hypertension OR hypertens* OR "high blood pressure") AND ("primary aldosteronism" OR hyperaldosteronism OR aldosterone OR "renal artery stenosis" OR "renovascular hypertension" OR "fibromuscular dysplasia" OR "obstructive sleep apnoea" OR "obstructive sleep apnea" OR pheochromocytoma OR phaeochromocytoma OR paraganglioma OR "Cushing syndrome" OR "Liddle syndrome" OR "Gordon syndrome" OR pseudohypoaldosteronism OR "glucocorticoid-remediable aldosteronism" OR "monogenic hypertension") AND (prevalence OR screening OR diagnosis OR sensitivity OR specificity OR "adrenal vein sampling" OR genetic* OR treatment OR "mineralocorticoid receptor antagonist" OR spironolactone OR amiloride OR "renal denervation" OR "continuous positive airway pressure" OR adrenalectomy OR therapy)

# Web of Science Core Collection

TS=(hypertension OR hypertens* OR "high blood pressure") AND TS=("primary aldosteronism" OR hyperaldosteronism OR aldosterone OR "renal artery stenosis" OR "renovascular hypertension" OR "fibromuscular dysplasia" OR "obstructive sleep apnoea" OR "obstructive sleep apnea" OR pheochromocytoma OR phaeochromocytoma OR paraganglioma OR "Cushing syndrome" OR "Liddle syndrome" OR "Gordon syndrome" OR pseudohypoaldosteronism OR "glucocorticoid-remediable aldosteronism" OR "monogenic hypertension") AND TS=(prevalence OR screening OR diagnosis OR sensitivity OR specificity OR "adrenal vein sampling" OR genetic* OR treatment OR "mineralocorticoid receptor antagonist" OR spironolactone OR amiloride OR "renal denervation" OR "continuous positive airway pressure" OR adrenalectomy OR therapy)

# Scopus

TITLE-ABS-KEY(hypertension OR hypertens* OR "high blood pressure") AND TITLE-ABS-KEY("primary aldosteronism" OR hyperaldosteronism OR aldosterone OR "renal artery stenosis" OR "renovascular hypertension" OR "fibromuscular dysplasia" OR "obstructive sleep apnoea" OR "obstructive sleep apnea" OR pheochromocytoma OR phaeochromocytoma OR paraganglioma OR "Cushing syndrome" OR "Liddle syndrome" OR "Gordon syndrome" OR pseudohypoaldosteronism OR "glucocorticoid-remediable aldosteronism" OR "monogenic hypertension") AND TITLE-ABS-KEY(prevalence OR screening OR diagnosis OR sensitivity OR specificity OR "adrenal vein sampling" OR genetic* OR treatment OR "mineralocorticoid receptor antagonist" OR spironolactone OR amiloride OR "renal denervation" OR "continuous positive airway pressure" OR adrenalectomy OR therapy)

# ClinicalTrials.gov

Condition/disease: hypertension. Other terms were iterated across primary aldosteronism; renal artery stenosis/renovascular hypertension; fibromuscular dysplasia; obstructive sleep apnea; pheochromocytoma/paraganglioma; Cushing syndrome; monogenic hypertension; mineralocorticoid receptor antagonist; renal denervation; CPAP; adrenalectomy. Combined registry records from ClinicalTrials.gov and WHO ICTRP contributed 19 records to the formal identification set.

# WHO ICTRP

Hypertension was combined iteratively with the same secondary-aetiology and cause-directed-therapy terms used for ClinicalTrials.gov. Registry duplicates were resolved during de-duplication. Combined registry records from WHO ICTRP and ClinicalTrials.gov contributed 19 records to the formal identification set.

# Supplementary citation and surveillance searches

Backward and forward citation searching was undertaken from included studies and relevant guidance. Society guidance was additionally retrieved from the Endocrine Society, AHA/ACC, ESC, ESH, ISH and WHO. A targeted surveillance update through 31 July 2026 identified major newly available guidance and pivotal late-indexed/new phase 3 evidence. Those records were used only to contextualise the discussion and were not added to the unchanged formal 580-record PRISMA flow or the formal GRADE cohort.
